# A neurocognitive interactive activation model of semantic priming in lexical decisions

**DOI:** 10.1038/s41598-026-58866-4

**Published:** 2026-06-20

**Authors:** Leo Sokolovič, Juraj Kukolja, Markus Hofmann

**Affiliations:** 1 Department of General and Biological Psychology, University of Wuppertal, 42119 Wuppertal, Germany; 2 Department of Neurology and Clinical Neurophysiology, Helios University Hospital Wuppertal, 42883 Wuppertal, Germany; 3 Faculty of Health, Witten/Herdecke University, 58488 Witten, Germany

**Keywords:** associative and semantic priming, leaky competing accumulators, interactive activation model, fMRI, Neuroscience, Psychology, Psychology

## Abstract

This study introduces the sequential read-out model (SROM) to investigate the strategic and decision-making contributions to the semantic priming effect in a lexical-decision task (LDT). We use behavioral and fMRI data from two experiments (*n* = 32 and 31), which factorially manipulated the association strength, semantic similarity, and stimulus-onset-asynchrony. Using a leaky competing accumulator layer for lexical decisions, the SROM successfully accounted for behavioral data and showed that switching from short to long stimulus-onset-asynchrony changed the decision-making mode from competitive to independent race regime. We then used the individually estimated SROM parameters to predict the interindividual differences in BOLD responses to targets. We found that lexical-orthographic evidence modulated BOLD amplitudes in the left lingual gyrus, suggesting that it maintains lexico-orthographic evidence in working memory, which is then used for lexical decisions. The decision threshold, inhibition parameters and top-down semantic excitation predicted activation differences in the left inferior frontal gyrus, suggesting it regulates the decision process. Decision noise and top-down semantic excitation were associated with the left angular gyrus, supporting its involvement in evidence accumulation. Overall, the SROM provided mechanistic interpretations of brain activations in the regions involved in the semantically primed LDT, while accounting for associative semantic and strategic factors.

**Supplementary Information:**

The online version contains supplementary material available at 10.1038/s41598-026-58866-4.

## Theoretical framework

### Semantic priming

Establishing brain-behavior associations in cognitive science and clinical neuropsychology faces two key challenges^1,2^. On the one hand, cognitive tasks depend on multiple cognitive processes^3^. On the other, these processes engage multiple brain regions^4,5^. For example, in a lexical decision task (LDT) participants must decide if the presented string of letters is a word or not. The semantic priming effect denotes the finding that lexical decisions are faster and more accurate, if a semantically related prime precedes the target^6–8^. There are two formal theoretical explanations of semantic priming^7,9^. According to localist models, processing of a word activates its node in the semantic network^10–12^. In priming, a prime’s activation spreads across the network, pre-activating potential targets in proportion to their association strength^11,13^. This pre-activation constitutes semantic priming. In contrast, in distributed models words are patterns of activation distributed over a range of concrete (e.g., properties^14^) or abstract processing units (features)^15–18^. The magnitude of the semantic priming effect is then proportional to the number and activation level of shared features between the prime and target^7,15^.

The primed LDT is used to investigate the structure of semantic memory in health and disease^7,8,19,20^, where deviations from the semantic priming effect indicate pathological processes^19,21–23^. Concretely, the dedifferentiation of the semantic store in Alzheimer’s disease leads to both increased and decreased priming effects^20,22^. For example, the loss of distinctive features results in hyper-priming by ‘repetition priming’ of semantic associates (i.e., cat and dog are treated as synonyms), while the disconnections between a target and its distinctive feature (e.g., stripes and tiger) attenuate priming by hindering spreading activation^19,22^. In contrast, vascular dementia patients struggle in choosing the correct response among competing alternatives^24,25^. Similarly, impaired semantic priming in Parkinson’s disease reflects impairments in performance and conflict monitoring as well as in response strategy adjustments^23^. Thus, semantic priming reflects semantic and executive processes.

### Dissociating associative and semantic priming

While localist and distributed models propose competing accounts of semantic representations^26^, behavioral data show priming due to both the spreading activation and the number of shared features. For example, shared features facilitate performance given a short (< 250 ms) stimulus-onset-asynchrony (SOA)^6,8^. However, at longer SOA (> 500 ms) strong semantic competition among words sharing many features prolongs visual word recognition. In contrast, priming due to spreading activation increases with the length of the SOA^7,27,28^. However, separating priming due to similar meaning (i.e., semantic priming) from priming due to word co-occurrence in word association tasks (i.e., associative priming) has been difficult, because associated words also share semantic features^6–8^.

This challenge was overcome by deriving semantic and associative relatedness measures from a large text corpus^3,13,29^. Specifically, Roelke et al.^13^ defined direct association (i.e., associative relatedness) to be present, when two words were more likely to co-occur in a sentence than on their own^29–31^. To define semantic similarity, they counted the number of common associates of two words^3,13,32^. These definitions enabled a factorial manipulation of semantic and associative priming^13^. For example, whereas *cold* and *hunger* often co-occur in sentences (i.e., are strongly associated), they have few common associates (i.e., are not semantically related [**associative priming**]). In contrast, *scale* and *range* can often be used as synonyms and seldom co-occur in sentences but have many common associates (**semantic priming**). A case of **strong association and semantic relatedness** is the pair *driver* and *car*. Finally, **neither strong association nor semantic relatedness** exists for *date* and *moose*. Roelke and colleagues found the effect of direct association at short and long SOA (associative priming), while the number of common associates facilitated performance only at short SOA (semantic priming)^6–8,13^. They thus provided empirical evidence for the distinction between associative and semantic priming. We therefore use the same definitions of associative and semantic priming. Furthermore, the semantic priming effect also reflects non-semantic factors, such as strategy use^7,21^. For example, expectancy generation uses the prime’s semantic context to anticipate targets, whereas semantic matching involves searching for a prime–target relation^7^. Strategy use can be reduced by using short SOA to prevent memory search and a low proportion of semantically associated words to attenuate expectancy generation^7,21^. Finally, the semantic priming effect also depends on how much lexical evidence one requires to make a lexical decision (i.e., the speed-accuracy trade-off)^33–35^.

## Models of lexical decision-making

Most distributed^15,16,18,27^ and localist models^10^ of semantic priming do not model the decision process^15,27,36^. For example, the Multiple Read-Out Model (MROM^10^) of visual word recognition simulated decision-making with multiple decision thresholds, one for the words’ orthographic unit activation, one for the overall lexical activation and a temporal deadline threshold. However, this decision mechanism leads to implausible response time (RT) distributions^34^. Instead, Wagenmakers and colleagues argued for the drift-diffusion model^37^(DDM; cf.^27^), a sequential sampling model of binary choice^38^. Sequential sampling models propose that noisy information is fed into a decision-making system, which accumulates evidence for the available alternatives^37,38^. The accumulation stops when either the word or the non-word decision threshold has been met, triggering the response^34,38,39^. Thus, the decision thresholds determine the speed-accuracy trade-off^34^. Wagenmakers et al. showed that the DDM better explained the LDT than the MROM (cf.^18,40^). However, Dufau et al.^33^ showed that, with appropriate input from the MROM, another sequential sampling model, the leaky competing accumulator (LCA^39^) can successfully model behavioral data. These studies highlight the need to incorporate decision-making process models into semantic priming models^15,36^, enabling principled explanations in both healthy and clinical populations, where identifying impaired processes informs diagnosis and treatment^1,21,41^.

## Functional neuroanatomy of semantic priming

The validity of cognitive models depends crucially on their neural plausibility; the demonstration of how or where in the brain the proposed cognitive processes take place^2,3^. Thus, to understand how different cognitive factors engender the semantic priming effect, their neural correlates were investigated^4,42,43^. The LDT was found to engage the occipital gyri, temporal areas (including the fusiform and lingual gyri), the inferior, middle and superior frontal gyri, the anterior cingulate cortex as well as the striatum^44–47^, i.e., regions supporting visual word processing.

The number of *common associates* (i.e., semantic priming) has been shown to modulate the processing in a number of brain regions, including the inferior frontal gyrus, the anterior cingulate cortex as well as the middle and inferior temporal lobes and posterior parietal regions^42,43,48,54^. *Direct association* (i.e. associative priming) also affect the left inferior frontal gyrus (LIFG), the left inferior temporal gyrus and the posterior parietal cortex^3,43,49^. The most notable difference between brain responses to direct and common associates is the involvement of the LIFG, which responded more strongly to prime-target pairs with few common associates but showed no activation differences for strong vs. no direct association^4,43^. The LIFG is proposed to resolve semantic competition between pre-activated long-term semantic representations^3,4^. This interpretation is supported by studies demonstrating that the LIFG receives information from the fusiform gyrus as early as 100 to 300 ms post-stimulus onset^50–52^. This enables it to select semantic representations by biasing the activation in the inferior temporal areas towards the desired semantic information^3,53^. However, the LIFG comprises functionally independent subregions. For example, the LIFG pars opercularis (Brodmann area 44, BA44) performs phonological processing, whereas the pars triangularis (BA45) partakes in conflict resolution and regulating the competition among activated semantic representations^42,53,55^. Finally, the pars orbitalis (BA47) is thought to retrieve semantic representations from a network of brain regions^4,17,42,53^. These studies suggest that cognitive control of semantic retrieval is coordinated by the LIFG in response to the competitive activation of common associates and direct associative word links in word processing areas.

Of the visual word processing regions, the anterior fusiform gyrus processes concrete words, whereas the posterior fusiform gyrus (including the visual word form area) represents visual word forms^56,57^ and interfaces them with higher-order properties (e.g., meaning)^58^. Apart from the fusiform gyrus, the increased activation of the left lingual gyrus in semantic priming reflects the working memory load^47,59^ and the strength of the direct association between primes and targets^48^. The lexical-semantic information is then integrated in the middle temporal gyrus^48,57^.

In the parietal cortex, the angular gyrus (AG) supports controlled retrieval, multimodal integration and prediction of orthographic and semantic information^60–64^, e.g., when reading sentences, unrelated words or accessing word meaning^65,66^. However, other work contests its role in semantic cognition and suggests that the AG, like the anterior cingulate cortex^45^, is involved in domain-general, associative and control processes^67^. For example, supporting problem solving, planning^60^ and decision-making^68^.

## Functional neuroanatomy of decision-making

In the LDT, an item’s representation, constructed by the orthographic and semantic processes, serves as the evidence for the word / non-word decisions^10,33,34,69^. In the context of sequential sampling models, evidence accumulation most likely involves areas supporting working memory, planning, directing attention and decision-making^70^, i.e., in the parietal and frontal cortices^5^. While the parietal cortices are more sensitive to incoming decision evidence, the frontal areas can maintain stable representations of decision alternatives and the current amount of evidence supporting them^5,61,71,72^.

For example, studies showed that the AG supports information accumulation at the intersection of memory and other cognitive systems^67,73,74^. This function reflects its preferential connectivity with other association regions and weak inputs from primary sensory regions^60^. For example, in memory tasks AG responded similarly to high evidence new and old stimuli, reflecting evidence accumulation^68,75^. Finally, evidence accumulation in the DDM is also associated with the fronto-parietal cortices and the striatum^5^.

However, the striatum^44,76^, together with the supplementary motor area^77^ and the anterior cingulate cortex^5,78^, has also been associated with the decision threshold and response bias. In these regions the BOLD response increases with decreasing decision threshold. Since the striatum is associated with evidence accumulation and the decision threshold, a division of labor between the striatum and the prefrontal cortex has been proposed^79^: whereas the prefrontal cortex maintains the possible response alternatives in working memory (e.g., word, non-word), the striatum maps the evidence to the available alternatives. This mapping combines the outputs of the perceptual^69^, lexical-semantic^10^ or mnemonic (i.e., previous learning^80–83^) processing with the information about the current stimulus’ task relevance (i.e., the top-down attention^70,84^). This combination determines the amount of evidence for each response alternative, which is assigned to their representations in the prefrontal cortex. Thus, striatal activity signals the regulation of the retrieval process and the transformation of the retrieved information into the decision evidence. Accordingly, difficult semantic retrieval leads to stronger striatal activation^44,79,85^. Finally, the involvement of the striatum in both, the evidence mapping (i.e., the processing of stimulus information) and in the setting of the response criterion suggest that the decision threshold might not be modulated only by the increasing time pressure (i.e., a temporal deadline^10^) but also by task difficulty and stimulus information^86–88^.

In summary, existing research suggests that the semantically primed LDT is supported by the left fusiform and lingual gyri (visual word recognition)^3,89,90^; the left middle and inferior temporal gyri (semantic processing)^3^; as well as the LIFG^3,53^, the left caudate nucleus^44,79^, the left supplementary motor area^77^ and the left AG^67,68,70^ (decision-making). Further, localist computational models (e.g., the interactive activation model (IAM)^3,12^) provided theoretical support for these empirical observations. However, most of these models lack a dedicated decision-layer.

## The sequential read-out model (SROM)

To address this, we build on the MROM^10^ and the Associative Read-Out Model (AROM)^10,12,29^, which extended the MROM by an associative layer to represent semantics and simulate associative spreading activation. These models are the basis of the new Sequential Read-out Model (SROM)^91^. Like its predecessors, it is an IAM^12^, in which processing units are organized in layers and in which processing units can excite and inhibit each other. The units in the early layers correspond to visual features and letters, while the later layers code higher-order information such as orthographic word forms, word frequency and semantic association strength^3,10,29^. In SROM, the latter reflects the cooccurrence of words in a large text corpus (cf. Methods). In contrast to complex and difficult to interpret large language models^92^, which use large embeddings and billions of parameters, our relatively simple language modeling approach is better suited for localist models. It provides a statistical definition of word co-occurrence which can define associations between the IAM’s word representations^93^. While the number of common associates defines semantic similarity between two words; the direct association strength is the likelihood of two words to appear in the same sentence. Using these definitions of semantic and associative relationships among words is well suited for IAMs because they enable them to have a distributed representation of meaning in a localist architecture. That is, their units simultaneously represent words and interpretable semantic features. Consequently, because common associates allow the activation to spread from the prime to the target, the IAMs can mimic distributed models and priming due to shared semantic features.

**Fig. 1:**
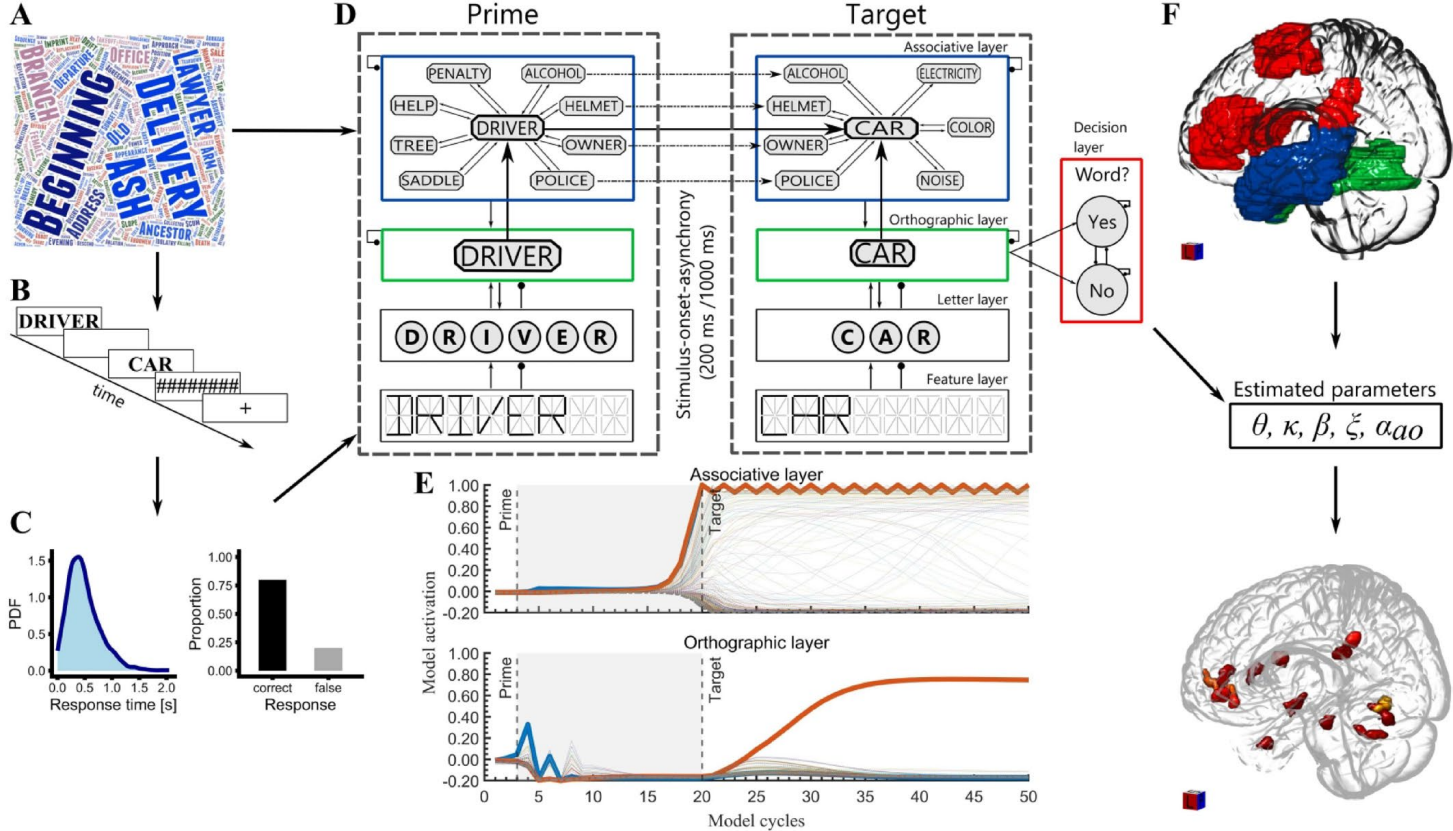
Model-based cognitive neuroscience approach. First, a large text corpus is used to select stimuli (**A**) for the experiment (**B**). Response times and choices are then collected from participants (**C**), who perform the lexical decision task (LDT). Panel (**D**) shows a schematic of the sequential read-out model (SROM). The model has five parallel-processing layers. The first layer represents visual features, which are combined into letters in the next layer. The letters then build words in the orthographic layer. There, the current stimulus’ representation inhibits other word units. In the associative layer, the direct association strength between the prime and the target regulates the magnitude of the target’s pre-activation by the prime. Additional pre-activation is generated by their shared associates. To model the lexical decision task, the SROM first processes the prime (left frame). During the following stimulus-onset-asynchrony spreading activation pre-activates the target. Next, the target is presented and processed by the model and its orthographic activation is read-out and transformed into the input for the decision layer. There, the decision-making process generates a word (Yes) or non-word response (No). Arrows indicate excitation and round line endings denote inhibition. Plot (**E**) shows the dynamics of the associative and orthographic layers for the stimulus pair driver-car. The prime is plotted in blue and the target in orange. The thin lines are the activations of the common associates. Note that the prime pre-activates the target, i.e., its activation increases before its onset. Finally, panel (**F**) shows that theoretical predictions constrain imaging analyses to selected regions-of-interest. The estimated model parameters are then used to predict and interpret their function.

The SROM initially represents the stimulus (prime or target) as a pattern of active and inactive visual feature units (see Fig. 1). Their activation excites the letter units in the next layer. The activated letters then excite the orthographic word units, which contain them and inhibit those which do not. The activation of the target’s orthographic unit constitutes the *word identification signal*^10^, which excites the target’s associative layer unit. In addition, the associative unit receives excitatory input via the spreading activation from its associates and direct excitation from the prime. Unlike in its predecessors, the activations of the processing units in the SROM are not reset after each stimulus, but only after each prime-target sequence. This allows the prime’s activation to persist during the SOA and pre-activate the target’s associative unit. A second novelty implements the proposed excitatory connection from the associative to the orthographic layer ($\:{\alpha\:}_{ao}$)^3^. It allows for top-down associative influences on orthographic processing by funneling a word’s associative activation to its orthographic unit. The SROM’s orthographic units therefore contain orthographic *and* associative information (cf. Figure 1), which enables the modeling of effects such as semantic priming and expectation generation in the primed LDT^3,7^.

Priming in the SROM results from the associative spreading of activation^3,29^. Concretely, the processing of a prime activates its unit in the orthographic and associative layers. In the associative layer, the prime’s activity is propagated to words associated with it^11^. These are the potential targets, which are pre-activated in proportion to the strength of their association with the prime^11,13^. This pre-activation of the associated nodes constitutes the semantic priming effect and facilitates the identification of the presented target as a word via the new top-down semantic excitation parameter.

### Decision-making as interactive evidence accumulation

An important limitation of the MROM and AROM is the missing decision-making layer^36^, which leads to inaccurate predictions of empirical RT distributions^34^. This shortcoming was addressed by modeling the decision process^33^ using the LCA^39^, which generates decisions via a competitive accumulation of decision evidence towards a decision threshold. The competition between decision units (i.e., response options) is governed by a set of parameters^39^. Like neurons, each unit experiences activation leakage (decay towards resting potential) and is additionally inhibited by other decision units. The strengths of the leak and mutual inhibition are governed by the *k* and *β* parameters, respectively. In two-alternative choice tasks, the effective differential leakage *λ* (i.e., *λ* = *k - β*) can be calculated^39^. This parameter measures the rate of the decision units’ activation decay. If *λ* = 0, the accumulation process is balanced, i.e., *k* = *β*, and corresponds to the DDM^38^, showing that it is a special case of the LCA. If *λ* ≠ 0, positive values are associated with slowing and negative values with accelerating the decision process^39^. The decision threshold (*θ*) determines the amount of evidence needed to end the decision process and initiate response preparation and execution. Finally, the evidence accumulation noise (*ξ*) represents decision-irrelevant processing and introduces stochasticity into the evidence accumulation.

While the LCA simulated empirically plausible RTs^33^, it was not integrated into the MROM and it was assumed that the IAM generates suitable input for the LCA. This assumption, however, should be empirically tested, because unlike the DDM, which estimates lexical input^34^, IAMs explicitly simulate the generation of lexical evidence.

## Modelling strategy

Like in our previous work^94^, we adopt a nested incremental modeling approach^95^ and integrate the LCA into the SROM to model LDT data from two published datasets^4,13^ which dissociated priming due to direct association and to common associates. While the SROM deterministically simulates visual, orthographic, and semantic processes driving lexical decisions^10,29^, the empirical inter-individual variability of RT is assumed to reflect differences in the strength of the semantic top-down modulation of orthographic processing and the stochastic decision process^33^. This is because in our data, stimuli from different priming conditions are balanced with respect to several orthographic variables (cf. Methods). The LCA contains two units to answer the question: ‘Is this a word?’. The ‘Yes’ unit accumulates the orthographic activity of the presented word^10^ and the ‘No’ unit accumulates evidence for the non-word response. The accumulation stops when the decision threshold is reached.

**Fig. 2:**
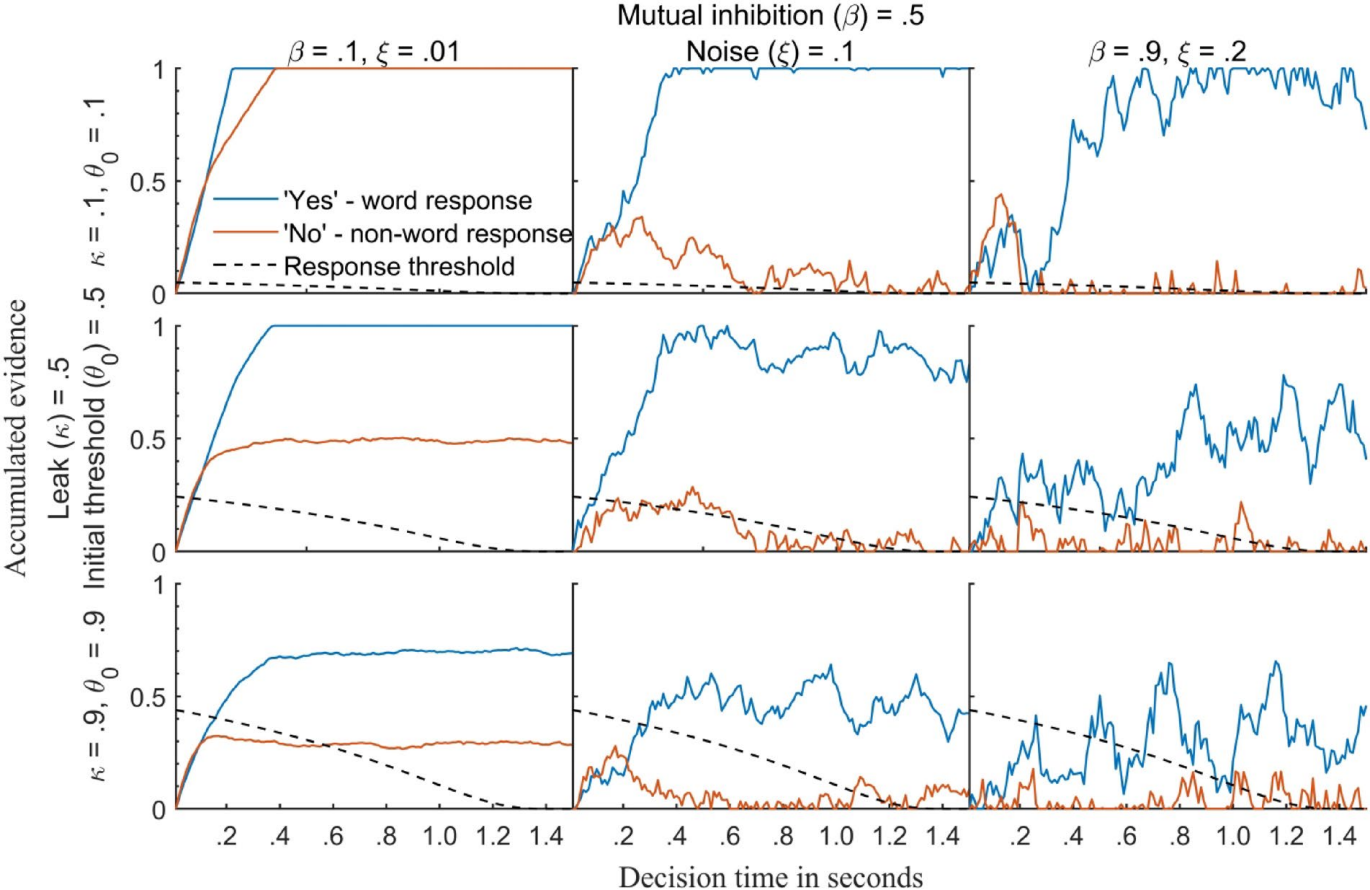
A sketch of the dynamics of a leaky competing accumulator model (LCA) with a collapsing threshold. This figure illustrates how LCA parameters influence the competition between response options to shape the decision-making process. Moving from left to right panels, increasing mutual inhibition (*β*) intensifies competition between the ‘Yes’ and ‘No’ units. This disproportionately slows down the accumulation of evidence for the weaker input (here ‘No’), making it less likely to reach the threshold first. Comparing panes from top to bottom, increasing leak (*κ*) values lead to increasing losses of accumulated lexical-decision evidence and cause both response activations to decay over time, reducing overall activation levels and delaying decisions. Increased noise (*ξ*) introduces variability into the evidence accumulation curves, increasing from left to right. When combined with a low response threshold, this can lead to fast but incorrect decisions driven more by random fluctuations than by input strength (first row). Increasing the response threshold (*θ*), from top to bottom, means that more accumulated evidence is needed to determine the response. This allows longer competition between alternatives, giving weaker but correct signals a chance to win out. However, it also prolongs the decision time.

We implement a collapsing decision threshold so that as time passes the amount of evidence required for a decision is reduced. The speed and extent of the reduction is determined by a word-familiarity measure, a general feeling that the presented string of letters is a word^96^. The final RT is thus influenced by the orthographic input strength, mutual inhibition between ‘Yes’ and ‘No’ units, activity decay, evidence accumulation noise and the decision threshold. The latter two parameters allow for occasional fast errors in the LDT^34^. An additional RT component is the non-decision time, representing motor preparation and execution^33,39^. The effects of varying the decision layer parameters are depicted in Fig. 2 (for mathematical details see Methods). We estimate the parameters for each participant separately to account for variability in the speed-accuracy trade-off. Additionally, for each participant, we also examined the effects of the SOA on the LCA parameters, since their values could depend on the time available for prime evaluation.

In sum, as Dufau et al.^33^, we model decision-making with an LCA module. Our approaches, however, differ in some important respects (see Fig. 1). First, we estimate parameter values by fitting the model to behavioral data from a LDT, which also manipulated the SOA (200 ms vs. 1000 ms). Second, because the LCA is integrated into an IAM, both LCA units receive time-varying input from the word’s orthographic unit. Third, we do not use noisy decision thresholds but allow the global orthographic and semantic activation to impact the decision threshold, much as lexical excitation affected the response criteria in the MROM^10^. Because decision thresholds are not random, the noisiness of decision-making is constrained to the process of evidence accumulation.

## Summary of the current study

The new SROM provides a mathematical framework, with which to assess the effects of the direct association strength, number of common associates, strategic and decision-making factors on semantic priming^2^. It does so by inheriting the abilities of the MROM and the AROM to simulate lexical-orthographic processing and associative spreading activation^11,29^. Furthermore, it adds the theoretically motivated decision layer^15,27,36^ and top-down semantic excitation^3^. The latter enables the word’s associative activation to impact its lexical-orthographic activation, which is fed to the decision units. A particular strength of the IAMs is that they represent complex neural computations at an abstract level, corresponding to the processing in brain regions and networks^3,97^. Thus, model-to-brain connections can be established by simulating cognitive processes with IAMs. To capitalize on this (cf. Figure 1), we use data which dissociates priming due to association strength and priming due to common associates. Moreover, the short and long SOAs also addressed the strategic contributions to the semantic priming effect.

The first aim of the present study is to assess the ability of the SROM to reproduce behavioral LDT data. This is an empirical test of the assumption behind the simulation results, which showed that the LCA simulated empirically plausible RT distributions and choice patterns^33,34^, given appropriate input from an IAM. To do so, the SROM’s free parameters are estimated separately for each participant and SOA duration. The individual estimates of the associative-to-orthographic layer excitation parameter and the LCA parameters (decision threshold, noise, leak, mutual inhibition and non-decision time) further enable us to study the strategic effects of the SOA on the decision-making process.

The second aim of our study is to leverage the SROM to provide mechanistic interpretation^3,98^ of neural correlates of the lexical and decisional contributions to the LDT. To do so, we use the estimated parameters to predict BOLD responses in a theoretically motivated set of brain regions^3^. Based on the studies we reviewed above and on the previous work on IAMs^3^, we hypothesize that the word identification signal will modulate the activity of the fusiform and lingual gyri^3,89^, with the LIFG pars orbitalis acting as the source of top-down semantic control ($\:{\alpha\:}_{ao}$ parameter)^3,42,53^. Top-down semantic excitation should also predict BOLD responses in other areas associated with semantic processing, such as the temporal cortices^3,90^ and the angular gyurs^61^. We also expected that the LCA’s decision threshold would be associated with the striatum and the prefrontal areas^77,78^. Since leak and mutual inhibition parameters control evidence accumulation, they should affect cognitive control and evidence accumulation regions (e.g., LIFG, supplementary motor area, fusiform and angular gyri)^5,68^. These areas should also be susceptible to the evidence accumulation noise.

## Results

We analyzed RTs of correct responses and error rates from two experiments using the same LDT (for details see the Methods section). Experiment 1 (*n* = 32) data were obtained from Roelke et al.^13^ and Experiment 2 (*n* = 31) data from the fMRI study by Roelke and Hofmann^4^. The 200 prime-target pairs for the LDT were assigned to eight conditions resulting from a full factorial design combining three factors: SOA (long [1000 ms], short [200 ms]), direct association strength (strong, none) and the number of common associates (many, few), i.e., semantic similarity. The stimulus selection controlled for important linguistic variables such as word length and frequency as well as the number of orthographic neighbors and ensured that there was no confounding of the associative and semantic contributions to semantic priming (for stimulus selection details see^13^).

### Observed and simulated response times and error rates

**Fig. 3:**
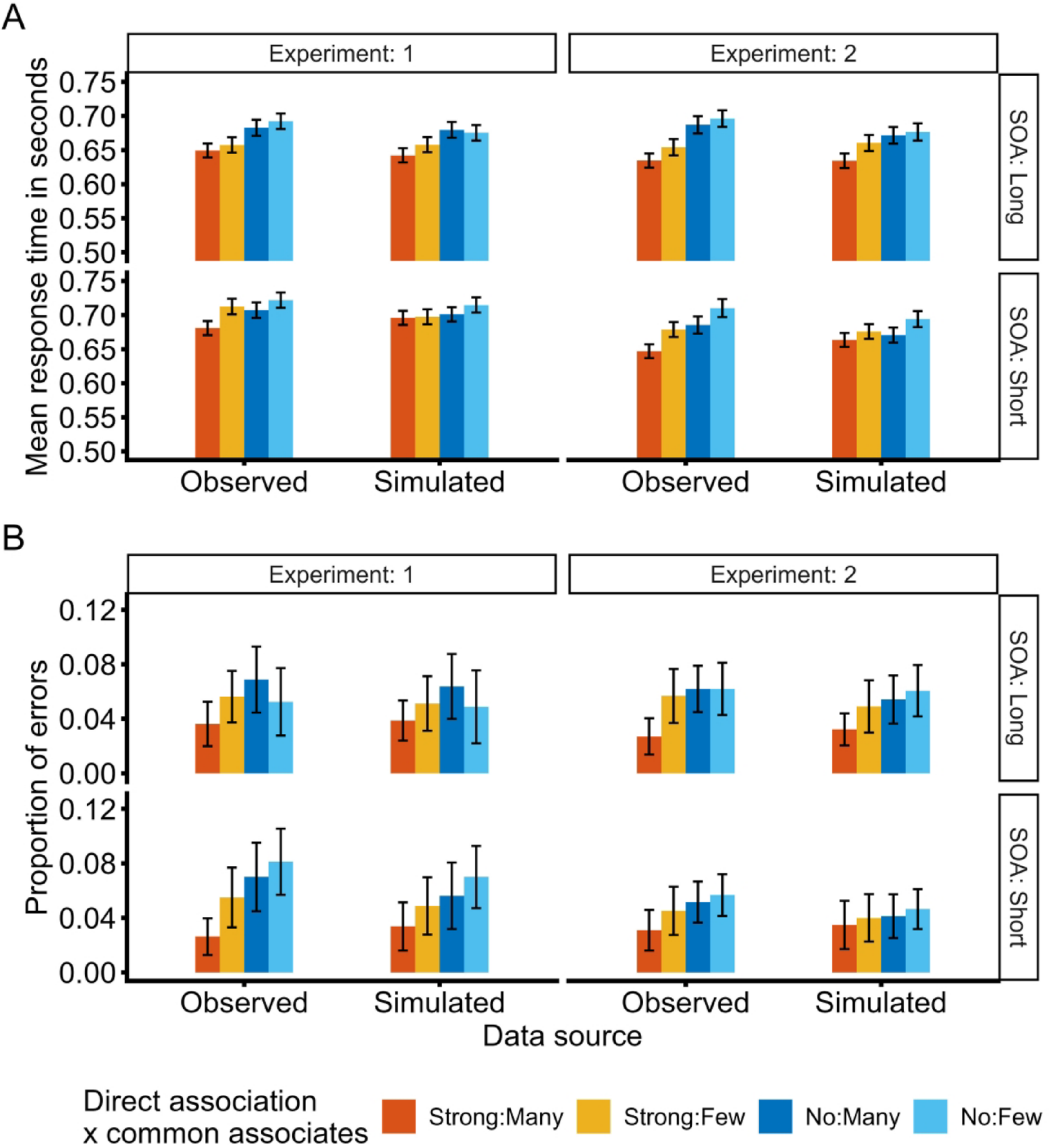
Comparisons of observed and simulated data. The figures show mean values of response times (RT; panel **A**) and error proportions (**B**) of the eight experimental conditions. The error bars denote 95% confidence intervals. SOA: stimulus-onset-asynchrony.

We first assessed the global fit across both experiments by calculating the correlations between the observed and simulated average RTs and the number of errors. We found a high correlation for RTs *r*_(61)_ = 0.98 (95% CI [0.98, 0.99], *p <* 0.001) and the number of errors *r*_(61)_ = 0.98 (95% CI [0.96, 0.99], *p <* 0.001). Next, we examined the ability of the SROM to reproduce the patterns of mean RTs and errors in the observed data. Supplementary Table 5 reports the mean and standard deviations of the RT for each experimental condition for simulated and observed data (see also Fig. 3). Supplementary Table 5 also lists the correlations between the observed and simulated average RTs across participants, indicating very good correspondence for both experiments (all *ρ >* 0.86, for the similarity of simulated and observed RTs distributions see supplementary Fig. 1).

Figure 3 and supplementary Table 6 show the observed and simulated error proportions. The SROM predicted error rates also showed good correspondence with the observed error rates (all rank correlations *ρ >* 0.46). Finally, Table 1 compares the observed and simulated priming effects and shows that the SROM replicated the priming effects in the observed data. The are, however, two exceptions. First, the RT semantic priming effect in the short SOA in Experiment 1 is only marginally significant (t(31) = -2.01, *p* = 0.053) in the simulated data. The second deviation is the non-significant reduction in the proportion of errors in the short SOA for the combined (associative and semantic) priming condition of the second experiment (t(30) = -1.33, *p* = 0.194). Thus, the SROM successfully captured the observed data.

**Table 1:** Priming effects in observed and simulated data. The effects were calculated by subtracting the average response times (RT) and error percentages (% error) from the mean RT and % error of targets with no direct and few common associates.

SOA		Associative		Semantic		Associative & semantic	
Experiment 1							
		Observed	Simulated	Observed	Simulated	Observed	Simulated
200 ms	RT	-10 (7)	-20 (8)	**-10 (6)**	-10 (6)*	**-40 (7)**	**-20 (7)**
	% error	-3 (1.3)	-2 (1.2)	-1 (1.2)	-1 (1.1)	**-2 (0.9)**	**-4 (1)**
1000 ms							
	RT	**-40 (7)**	**-20 (6)**	-10 (7)	0 (6)	**-40 (6)**	**-30 (5)**
	% error	0 (1.1)	0 (1.3)	2 (1.1)	2 (1.1)	-6 (1.2)	-1 (1.1)
Experiment 2							
200 ms	RT	**-30 (10)**	**-20 (6)**	**-30 (11)**	**-20 (6)**	**-60 (9)**	**-30 (6)**
	% error	-1 (1)	-1 (0.9)	-1 (0.7)	-1 (0.7)	**-3 (0.8)**	-1 (0.9)
1000 ms							
	RT	**-40 (7)**	**-20 (5)**	-10 (8)	-10 (4)	**-60 (11)**	**-40 (8)**
	% error	-1 (1)	-1 (0.8)	0 (0.9)	-1 (0.8)	**-3 (1.2)**	**-3 (0.8)**
All priming effects for RT are in ms and for % error in percent. For all entries marked in bold the respective t-tests were significant at *p* < 0.05. *p = 0.530							

### SROM’s free parameters and behavioral data

Next, we analyzed SROM’s free parameters. Parameter definitions and the descriptive statistics of the fitted values are reported in supplementary Table 3. All pairwise correlations (*df* = 30 in experiment 1 and 29 in experiment 2) between the parameters are reported in the supplementary Tables (7 to 10). In the short SOA conditions in both experiments, we found significant correlations between the decision threshold (*θ*) and the leak parameter *κ* ($\:{\rho\:}_{Exp.\:\:1}$ = -0.64, $\:{\rho\:}_{\:Exp.\:\:2}$ = -0.64), *θ* and the mutual inhibition parameter *β* ($\:{\rho\:}_{Exp.\:\:1}$ = -0.77, $\:{\rho\:}_{Exp.\:\:2}$ = -0.62) as well as between *θ* and the associative to orthographic excitation strength ($\:{\rho\:}_{Exp.\:\:1}$ = -0.58, $\:{\rho\:}_{Exp.\:\:2}$ = -0.70). For the long SOA only the correlation between *θ* and *κ* was significant in both experiments ($\:{\rho\:}_{Exp.\:\:1}$ = -0.70, $\:{\rho\:}_{Exp.\:\:2}$ = -0.55). These results show strong correlations among the decision-layer parameters, which control the evidence accumulation process. For the fMRI analyses, we therefore used the effective differential leakage parameter, which did not significantly correlate with any other parameter (see supplementary Tables 9 and 10 as well as 11 and 12 for SPM’s cosine similarities).

**Fig. 4:**
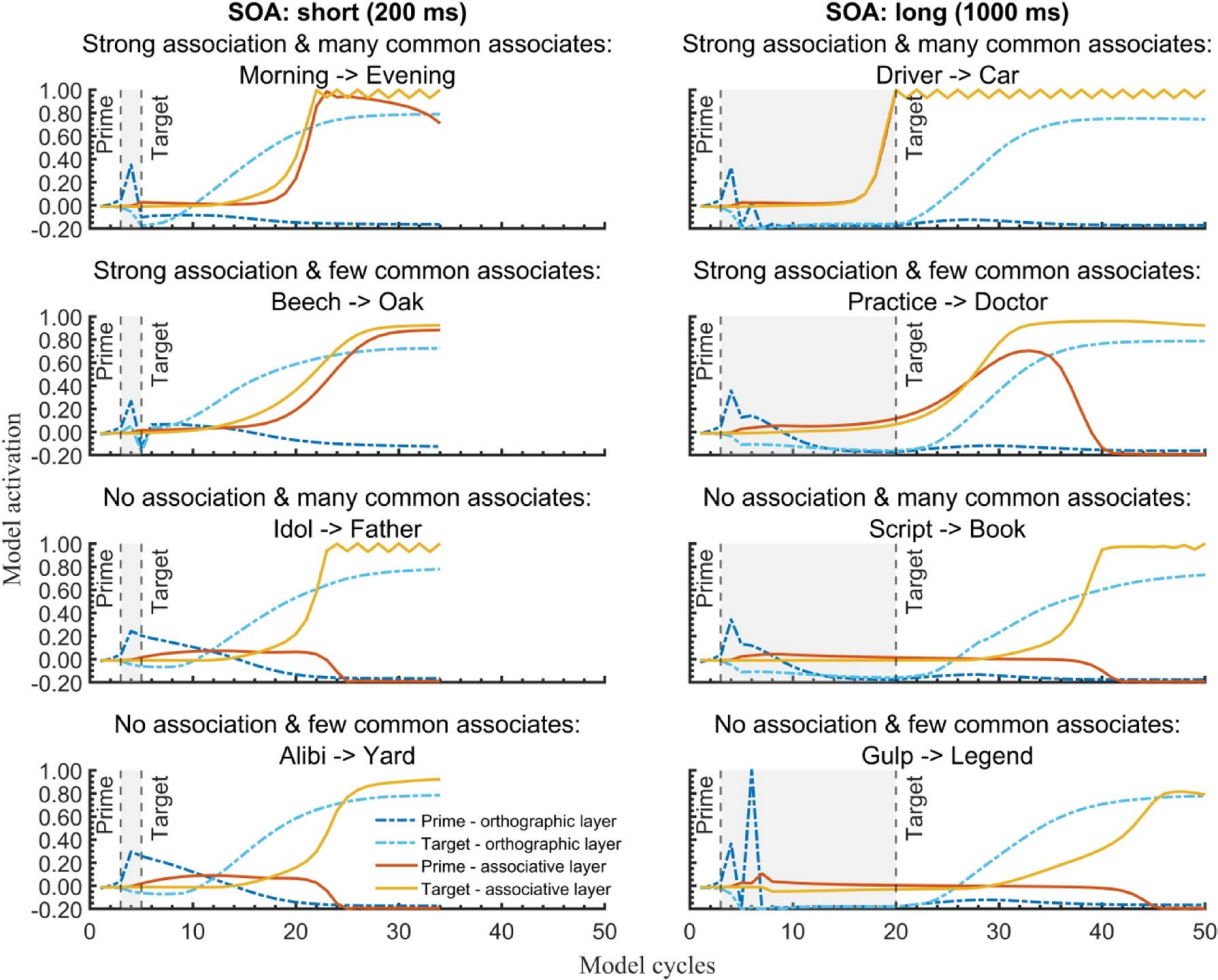
Example orthographic activations of stimuli from the eight experimental conditions. The time courses show reduced and delayed associative activation of the targets with decreasing association strength and few common associates. The figure is based on the model with the associative to orthographic excitation (*α*_*ao*_) set to 0.04. This was the median value across both experiments. Note: SOA = stimulus-onset-asynchrony.

Supplementary Tables 7 to 10 also report the correlations between the model parameters, the RTs and the proportion of correct responses. None of these correlations were significant. Thus, we found no evidence for a direct relationship between individual parameters and the observed RTs and accuracy. To further explore the semantic priming effect in the SROM, we plotted the activations of primes and targets in the orthographic and associative layer. As expected, Fig. 4 shows that during the long SOA the associative activation of the targets with strong association and many common associates with their primes builds up and remains high throughout the decision period. The magnitude of pre-activation also varies with the strength of direct association, the number of common associates and the length of the SOA. In the orthographic layer, the lack of mutual excitation and strong mutual inhibition (see supplementary Table 1) lead to smaller differences between the activations of targets from different conditions.

### The effect of the SOA on the decision layer parameters

To investigate strategic shifts in decision parameters, we compared the values of the decision threshold, the leak and the mutual inhibition parameters in the SOA conditions (short - long). In Experiment 1, the Wilcoxon signed rank tests showed significant differences between the values of the leak parameter (*κ*) (*Med*_*difference*_ = 0.11, 95% CI [0.01, 0.25], *p* = 0.034, *r* = 0.37), the mutual inhibition (*β*) (*Med*_*difference*_ = 0.13, 95% CI [0.05, 0.23], *p* = 0.003, *r* = 0.52) and the decision threshold (*θ*) (*Med*_*difference*_ = -0.04, 95% CI [-0.07, -0.02], *p* = 0.001, *r* = 0.58). The comparisons were also significant in Experiment 2. Specifically, the Wilcoxon signed rank tests showed significant differences between the values of *κ* (*Med*_*difference*_ = 0.16, 95% CI [0.04, 0.28], *p* = 0.004, *r* = 0.52), *β* (*Med*_*difference*_ = 0.08, 95% CI [0.00, 0.19], *p* = 0.047, *r* = 35) and *θ* (*Med*_*difference*_ = -0.05, 95% CI [-0.07, -0.02], *p <* 0.001, *r* = 0.61). These results showed that the short compared to long SOA duration was associated with increased inhibition parameters and reduced decision thresholds. This pattern suggests a change from stronger to weaker competition between the decision-layer units, with the decision threshold also changing to regulate the speed-accuracy trade-off.

### Neural representation of the word identification signal

In the fMRI data analysis, we first investigated the effect of the parametric modulator in the left fusiform, lingual as well as inferior and middle temporal gyri. The analysis showed that the targets’ orthographic activation led to a significant activation of the left lingual gyrus (cluster size 5 voxels, peak voxel: *F*_(1,30)_ = 24.91, $\:{p}_{FWE}=0.043$, MNI coordinates: -10 -74 0). No other significant results were found.

### Individual differences in SROM’s parameters modulated the BOLD response in orthographic, semantic and decision-related areas

After identifying the source of the word identification signal, we used the SROM’s free parameters to predict the BOLD responses in a set of left hemispheric regions of interest containing brain areas responsible for orthographic, semantic and decision-making processes. The fMRI analyses used the effective differential leakage parameter (*λ* = *κ* – *β*) because of strong multicollinearity between the decision threshold (*θ*), leak (*κ*) and mutual inhibition (*β*).

**Fig. 5:**
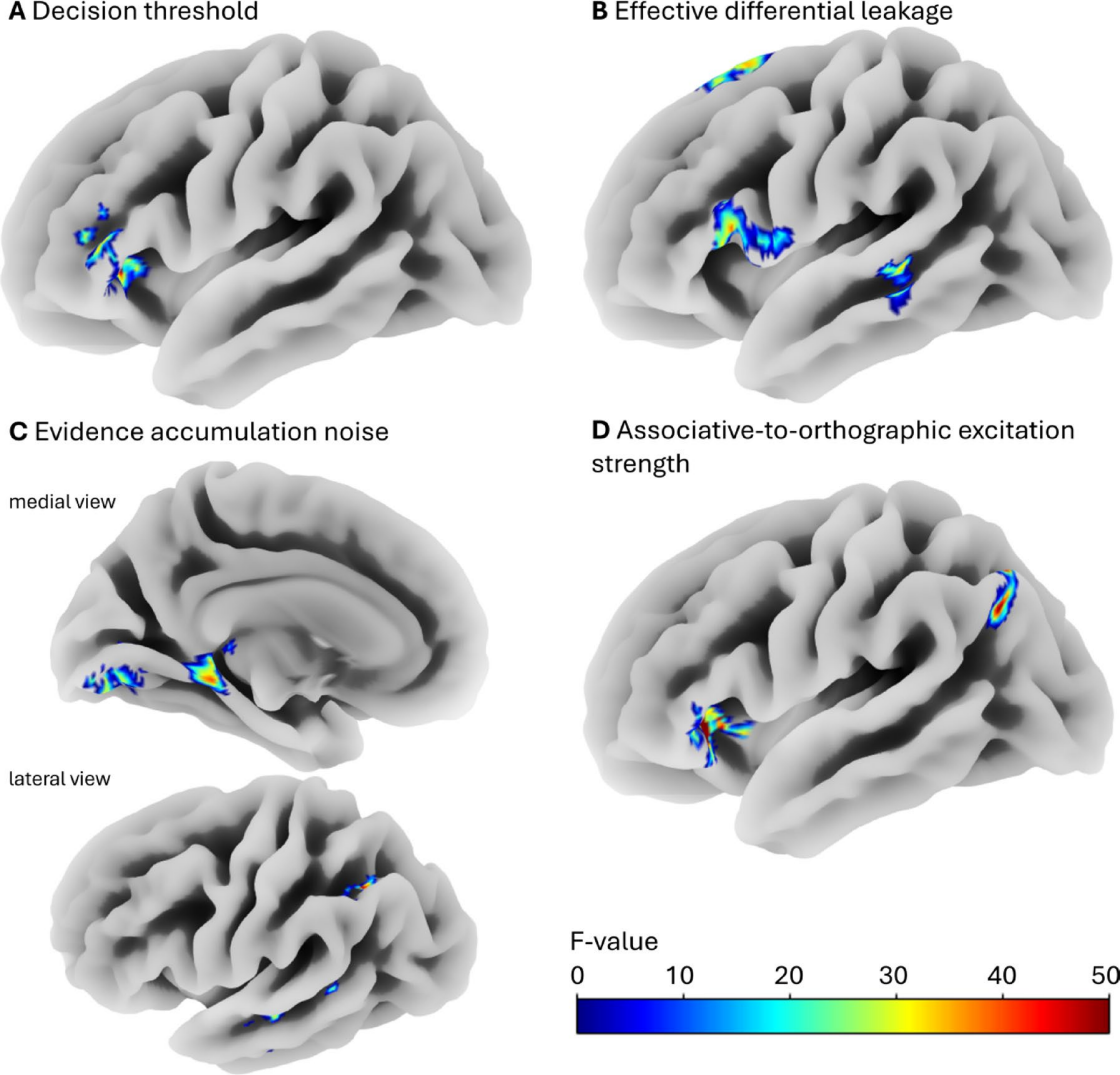
Brain areas significantly associated with the SROM’s free parameters. In C both a medial and a tilted lateral view are shown to display the activations in the lingual and angular as well as temporal gyri. The left inferior frontal gyrus (LIFG) was modulated by differences in the decision threshold (**A**), the top-down semantic excitation (**D**) as well as the effective differential leakage (**B**). This specifies the LIFG’s role in semantic control to both top-down semantic facilitation of orthographic processing as well as to balancing the speed and accuracy of decisions by setting the inhibition levels and response thresholds. The effective differential leakage also involved other regions associated with cognitive control such as the posterior middle temporal gyrus (affecting semantic representations) and the supplementary motor area (delaying responses). The differences in the BOLD response in the angular gyrus were associated with the top-down semantic excitation (semantic retrieval; panel D) and with the noise parameter (evidence accumulation; panel C). The latter also influenced the inferior and middle temporal as well as the lingual gyri, which reflects its deleterious effect on evidence generation and maintenance.

The results are reported in Fig. 5; Table 2, which also contains the automatic anatomic labels (AAL3) results. They show that stronger associative-to-orthographic excitation ($\:{\alpha\:}_{ao}$) was associated with stronger BOLD responses in the AG and the LIFG pars orbitalis as well as weaker activation of the LIFG pars triangularis. This suggests that $\:{\alpha\:}_{ao}$ was associated with recall of semantic information, which facilitated evidence accumulation (see Fig. 5 panel D).

**Table 2:** fMRI results: neural correlates of the SROM’s free parameters.

parameter	cluster size	brain area	effect direction	$\:{\mathrm{F}}_{\left(\mathrm{1,232}\right)}$	MNI coordinates
decision threshold	132	LIFG pars triangularis	$\downarrow$	40.09	-44 38 6
	66	LIFG pars orbitalis/pars triangularis	$\uparrow$	54.20	-30 32 − 2
effective differential leakage (leakage – mutual inhibition)	79	caudate nucleus	$\uparrow$	58.47	-18 0 18
	60	caudate nucleus	$\uparrow$	41.13	-14 24 6
	76	middle temporal gyrus	$\uparrow$	52.01	-52 -34 4
	132	LIFG pars triangularis	$\downarrow$	42.63	-52 22 16
	119	supplementary motor area	$\uparrow$	39.21	-10 10 72
evidence accumulation noise	79	angular gyrus	$\downarrow$	100.97	-30 -56 32
	81	inferior temporal gyrus	$\uparrow$	45.25	-46 -50 -10
	79	middle temporal gyrus	$\downarrow$	44.39	-50 -28 -14
	201	lingual gyrus	$\downarrow$	51.06	-20 -72 -2
	70	lingual gyrus	$\uparrow$	42.92	-14 -40 -8
associative-to-orthographic excitation	128	LIFG pars orbitalis	$\uparrow$	88.70	-30 28 − 4
	103	angular gyrus	$\uparrow$	58.84	-38 -66 46
	79	LIFG pars triangularis	$\downarrow$	33.76	-42 30 − 2

Note: all regions are in the left hemisphere; Left inferior frontal gyrus (LIFG). All peak voxels $\:{p}_{FWE}<\:0.05$.

Higher initial thresholds were associated with reduced BOLD responses in the LIFG pars triangularis and increased BOLD signal in the LIFG pars orbitalis. Thus, higher decision thresholds (i.e., prolonged decision time) were also associated with increased retrieval of semantic information (see Fig. 5 panel A). Like the decision threshold, the effective differential leakage parameter (*λ*, see Fig. 5 panel B) decreased the activation of the LIFG pars triangularis by decelerating the evidence accumulation process. Higher values of this parameter (i.e., stronger leak) further increased the activation of the supplementary motor area and areas representing and mapping decision evidence to the available response alternatives (i.e., middle temporal gyrus and the caudate nucleus).

Finally, increased evidence accumulation noise *ξ* was associated with reduced BOLD responses in the AG, the middle temporal gyrus and the posterior lingual gyrus region modulated by the orthographic activation. In contrast, increased BOLD responses in the visual word form area (MNI coordinates: -46 -50 -10) and the anterior lingual gyrus (see Table 2; Fig. 5 panel C). Thus, evidence accumulation noise affected both the representation of the word identification signal and the evidence accumulation process.

## Discussion

This study answers the call to provide a comprehensive account of the LDT by including a decision-making module in models of semantic priming^15,36^. We thus modified the AROM^29^ in three ways thereby creating the sequential read-out model (SROM). First, the SROM processes prime-SOA-target sequences, enabling primes to pre-activate targets during the SOA^3,11,13^. The pre-activation’s magnitude is determined by text corpus-derived language-modeling metrics, which can dissociate priming due to the association strength and the number of common associates^13^. The SROM uses these metrics to set the resting levels and connection weights in the associative and orthographic layers. Second, a connection from the associative to the orthographic layer models semantic strategic top-down processes^3^. In the SROM, semantic priming thus stems from its structural properties, spreading associative activation (see Figs. 1 and 4), and the top-down semantic modulation of orthographic processing via the associative-to-orthographic excitation ($\:{\alpha\:}_{ao}$). Third, the target’s orthographic activation drives the LCA^39^ layer to produce lexical decisions. With these modifications the SROM models the associative, semantic, strategic and decision-making aspects of the primed LDT. The first aim of the study was to assess the ability of the SROM to account for behavioral LDT data. In the next step, model-to-brain connections^2,3^ were established by using the fitted parameters to predict BOLD responses in brain areas involved in the primed LDT.

We found that the SROM accurately modelled empirical data (see Fig. 3) and generated RT distributions similar to the observed ones^34^. This supports the theoretical assumption^33^ that the word identification signal^10^ enables the LCA to reproduce observed data. Indeed, $\:{\alpha\:}_{ao}$ (0.06) was on average stronger than excitation within the associative layer ($\:{\alpha\:}_{aa}$ = 0.03), but weaker than orthographic-to-associative excitation ($\:{\alpha\:}_{oa}$ = 0.09), confirming that lexical decisions in healthy participants are primarily driven by orthographic information (cf.^29^). This information is accumulated in the LCA-layer until it reaches a decision threshold, which depends on the decision context^44^, i.e., the momentary global orthographic and associative activation. This is a sense of word-likeness^96^, which progressively reduces the decision threshold, inducing a temporal deadline. Using a dynamic threshold, we could remove the MROM’s global activation and temporal deadline thresholds while more accurately accounting for RT distributions^10,34,94^. Note that the decision threshold’s adaptation mechanism is task specific and intended to examine the effects of different priming conditions. By definition, its value will decay faster for targets primed with semantically related primes because of the direct pre-activation or the pre-activation of the common associates. However, the mathematical formulation could be more generally used to study lexical decisions in visual single-word recognition. For instance, one could compare the IAM with our collapsing-thresholds approach with an IAM using the trial-to-trial adaptive decision threshold implemented by Dufau and colleagues^33^. That is, the threshold’s value will decay faster for words with more direct associates (which potentially share many common associates) because of a greater buildup of global semantic activation^10,29,54^. A more general test of our threshold mechanism could, for instance, investigate whether higher word-likeness due to higher orthographic neighborhood leads to a faster decay of the decision threshold, compared to lower word-likeness. Indeed, standard single-word recognition is most closely like our data from the short SOA, weak direct association strength and no common associates condition. For this minimal priming condition, supplementary Fig. 2 shows that the decision threshold’s decay varies across targets, reflecting differences in the connection strengths in their respective orthographic and associative networks.

**Fig. 6:**
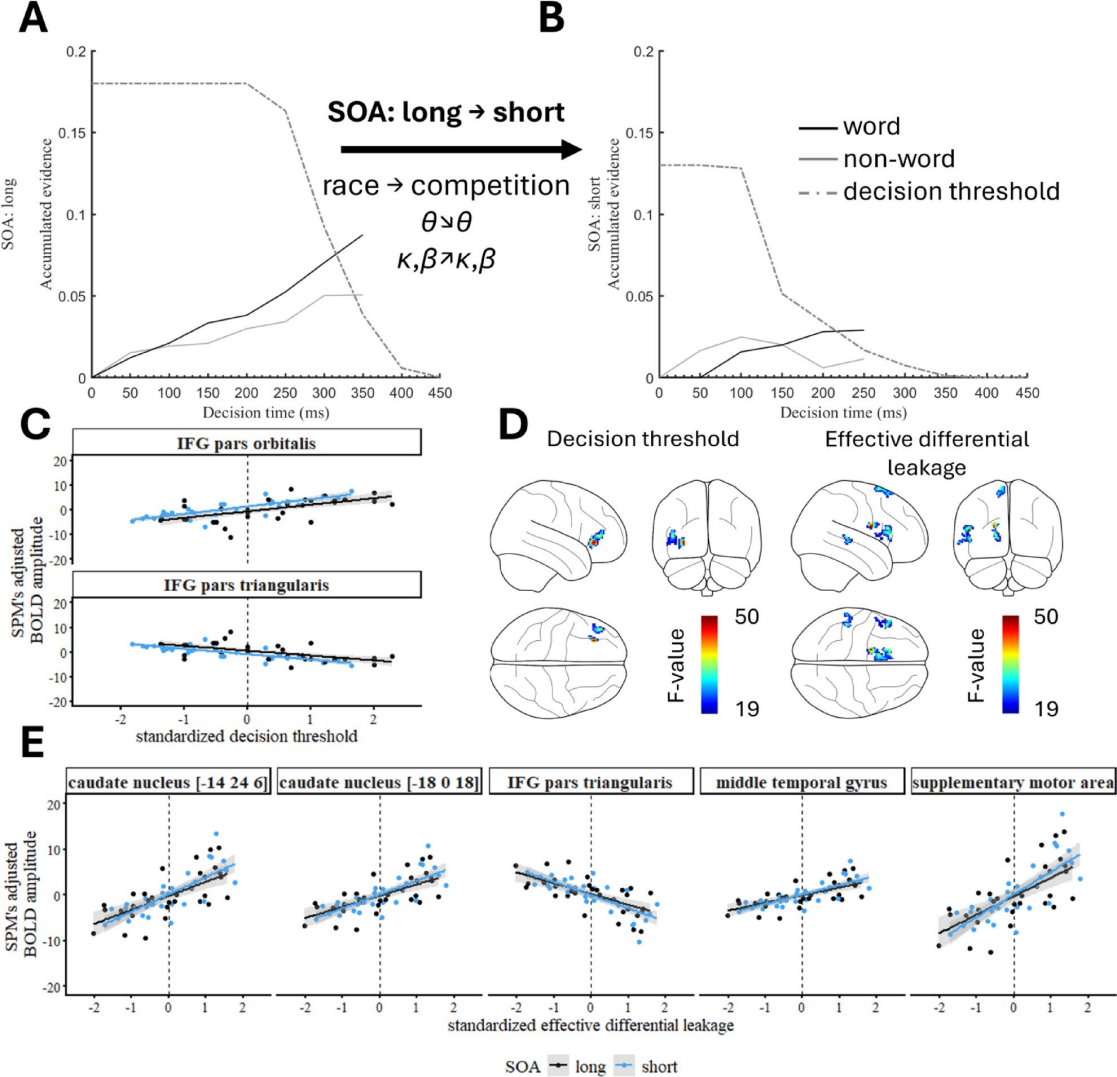
Effects of the stimulus-onset-asynchrony (SOA) on the decision-making parameters. Shortening of the SOA increased leak (*κ*) and mutual inhibition (*β*) but decreased the decision threshold (*θ*). This corresponds to a transition from an independent race-like (**A**) to a more competitive decision-making regime (**B**). Whereas the accumulators can race towards the threshold in A (diverging lines), in B the word response must rely on mutual inhibition and outcompete the non-word response (crossing accumulation lines). Panel **D** shows the regions associated with the parameters governing the decision-regime switch and panels **C** and **E** the relationships between BOLD responses and model parameters. Panel **C** shows that higher decision thresholds were associated with stronger activation of the left inferior frontal gyrus (IFG) pars orbitalis and reduced activation of the IFG pars triangularis. This suggests that participants who required more evidence to decide also retrieved more semantic information (pars orbitalis). Panel **E** shows analogue plots for the effective differential leakage parameter (*λ* = *κ* - *β*). Strong *λ* (high leak and low mutual inhibition) was associated with higher activation in the caudate nucleus and supplementary motor area, indicating increased cognitive control of the evidence accumulation process. Moreover, the attenuation of the IFG pars triangularis activation also suggests that this region determines the threshold and evidence accumulation regulation. Higher *λ* was also associated with stronger lexicosemantic search as indexed by the increased middle temporal gyrus activation.

Analyzing the effects of the SOA duration on the decision threshold (*θ*), leak (*κ*) and mutual inhibition (*β*) parameters, we found that the SOA induced strategic changes in these parameters, while not affecting accuracy (see supplementary Table 6). Compared to long SOA trials, short SOA trials were characterized by stronger leak and inhibition parameters but lower decision thresholds. This suggests that during short SOA the prime’s activation had little time to spread to the target, leading to a relatively weak decision-layer input. The strong competition in the decision layer decelerated the evidence accumulation process, allowing the yes/word accumulator to win. However, to decide within the response window, the decision threshold must have been lowered as well. In the short SOA, decision thresholds and semantic top-down excitation were negatively correlated, showing that participants with stronger top-down excitation required less information to make lexical decisions. In the long SOA, spreading activation pre-activated the targets, resulting in stronger inputs for the word response unit. This led to higher decision thresholds, while leak and mutual inhibition parameters were reduced. Thus, the effect of SOA change was a strategic switch from a competitive to a more independent decision process (cf.^99^ and Fig. 6). Accordingly, the participants could expect faster evidence accumulation in favor of the word and stronger inhibition of the non-word unit. The decision threshold could thus be set higher and allow the fastest accumulator to win. However, while the accuracy was constant, the responses were slower in the short compared to long SOA (see supplementary Tables 5 and^100^). Thus, while the decision threshold determined the amount of evidence needed for a decision, the duration and the dynamics of the decision phase were affected more by the leak and mutual inhibition parameters, thereby accounting for longer RTs at shorter SOAs. In sum, the analyses of the SROM’s estimated parameters show the rich dynamics of the decision-making process in the primed LDT and identify a third strategic factor in task performance - the shifts in the decision layer’s parameter values, which complement expectancy generation and semantic matching^7^.

Our second aim was to use the SROM to mechanistically interpret the activations in brain regions supporting the LDT. We thus regressed the BOLD responses in brain areas commonly involved in the LDT on the SROM’s parameters. The analysis showed that the word identification signal modulated the BOLD response in the left lingual gyrus. It is known that this region processes visually presented words^47^ and shows a semantic priming effect^48,56^. However, unlike the visual word form area, which creates the visual word form of a current item^90^, the lingual gyrus maintains this representation in working memory^59^, thereby facilitating lexical decisions^47,59^. This corresponds well to the orthographic activation’s role in the SROM as the information driving lexical decisions.

The associative-to-orthographic excitation parameter, $\:{\alpha\:}_{ao}$, was expected to affect the LIFG and the AG^3^. Indeed, stronger $\:{\alpha\:}_{ao}$ decreased BOLD responses in the LIFG pars triangularis and increased BOLD responses in the pars orbitalis. These results suggest that $\:{\alpha\:}_{ao}$ increased recall from semantic memory (pars orbitalis), while also increasing semantic competition (pars triangularis)^4,42,53^. Stronger $\:{\alpha\:}_{ao}$ also led to stronger activation of the AG. AG has been associated with numerous cognitive processes, such as attention, multidomain information integration, semantic processing, memory recall^60,61,65,67^ and evidence accumulation^5,68,70^. Our results suggest that AG’s activation reflects the top-down semantic excitation of orthographic processing, i.e., the retrieval of lexical knowledge, which facilitates the accumulation of decision evidence. Note also that the evidence accumulation noise (*ξ*) decreased BOLD responses in the AG, however, more ventrally. This is in line with previous studies suggesting functional subregions of the AG, with ventral AG associated with general information integration and dorsal AG associated with semantic top-down control processes^61,64,101^. Thus, our results implicate the AG in both attention-guided retrieval of semantic information and evidence accumulation.

The evidence accumulation noise (*ξ*), i.e., task irrelevant activity^39^, was further expected to affect other decision-making areas. It indeed attenuated the activations in the region of the lingual gyrus modulated by orthographic activation (i.e. the decision evidence buffer) and of the middle temporal gyrus (MTG), a region central to semantic processing^3,102^. *ξ* also increased BOLD responses in the visual word form area^90^ and the anterior lingual gyrus. These results suggest that *ξ* affects the decision process directly by injecting noise into the evidence accumulation process (AG^5,70^) and indirectly by affecting the ventral occipito-temporal cortex thereby impeding the generation and maintenance of the target’s representation in working memory^59,89^.

Higher initial decision thresholds attenuated BOLD responses in the LIFG pars triangularis, suggesting that this region takes into account the SOA duration and the global lexical-semantic activation to set the initial decision threshold^53,60^. Higher decision thresholds were also associated with increased BOLD amplitudes in the pars orbitalis. This indicates that the accumulation process was prolonged in favor of semantic retrieval^42,53^. Contrary to our hypotheses and findings from the DDM^77^, the decision threshold did not affect the supplementary motor area or the striatum. However, we did find that both areas were associated with higher effective differential leakage (*λ*)^39^, i.e., the difference between the self-inhibition (*κ*) and mutual inhibition (*β*). Increased striatal responses signal an attenuated mapping of decision evidence to the response alternatives^44,79^, while increased BOLD amplitudes in the MTG and LIFG indicate controlled inhibition of the response alternatives^5,53,64,101,103^. Finally, higher *λ* also inhibited response preparation in the supplementary motor area^104,105^. Overall, this suggests that in the brain, as in the LCA, decision threshold and the regulation of evidence accumulation (*λ*) are distinct. While the former indexes the amount of evidence needed to respond, the latter determines the speed of evidence accumulation (cf. Figure 2).

The SROM’s localist architecture offers a principled interpretation of the neurocognitive processes involved in the LDT and can be used to mechanistically understand neurocognitive processes^26^. This is in stark contrast to deep neural networks (e.g., large language models^106^) for which one must first explain how the hidden units map to cognitive representations^107^ before it can be used to interpret the brain. Capitalizing on the SROM’s interpretability, a key future application is the study of impairments of language processing, such as dyslexia, aphasia or dementia. Currently, the SROM assumes unimpaired orthographic representations, but it could be used to test the prediction that dyslexia affects the orthographic and not associative-semantic layer^41^. Indeed, strong semantic-to-orthographic excitation has been shown to compensate impaired reading in dyslexia^108^. Alternatively, both dyslexia and aphasia could affect the decision-making stage^41^. Because SROM allows selective lesioning of orthographic, associative and decision-making processes, one can specify which aspects of representation and selection are affected in each disorder. SROM is also a formal framework in which differences between Alzheimer’s disease and vascular dementia can be studied. The two pathologies are thought to differentially affect the lexical-semantic system and the executive control of memory retrieval^19,20,24,25^. Specifically, while Alzheimer’s disease primarily impairs semantic memory^20,22,24,25^, vascular dementia affects monitoring and cognitive control of lexical retrieval and lexical decision-making^24,25^. Thus, Alzheimer’s disease should correspond to weakened connection weights and mutual inhibition in the SROM’s associative layer. In contrast, vascular dementia should affect the extraction of lexical evidence (e.g., the slope of the sigmoid function, associative-to-orthographic excitation strength) and the decision-layer parameters, affecting their values and the SOA related strategic changes.

The Sequential Read-Out Model (SROM) contains all the major stages of visual word recognition, simulates the spreading activation theory^11^ of semantic priming and uses orthographic-associative activations to drive lexical decisions. It successfully modelled behavioral data from two experiments and provided insight into lexical decisions by revealing strategic changes in the decision threshold and the regulation of evidence accumulation. Concretely, it suggested that limited spreading activation during the short SOA was accompanied by a competitive decision-making regime. At the long SOA, however, the strong pre-activation of the target was joined by a race oriented decision-making regime, in which decision units are more independent. The participants achieved their optimal performance by switching between these two decision modes. SROM’s free parameters also predicted interindividual differences in the BOLD response in areas responsible for cognitive control of semantic processing in lexical decisions. Specifically, the LIFG emerged as a central area which sets decision thresholds, regulates evidence accumulation and determines the strength of semantic top-down excitation. Semantic retrieval and evidence accumulation noise also affect the left AG. Finally, we found that the left lingual gyrus maintains the word identification signal in visual working memory.

### Methods

#### Ethics statement

No ethics approval was obtained because the data used in this study is non-sensitive and completely anonymized. Consent to participate is not applicable to the current study. However, informed consent was obtained from all individual participants included in the original studies^4,13^.

### Behavioral methods

#### Subjects and data

We fitted the SROM to data from two previously published experiments. In Experiment 1^13^, 32 participants performed a lexical decision task (12 male, 20 female, *M*_*Age*_ = 26.69, range: 20–40 years). In Experiment 2^4^, another set of 32 participants (12 male, 20 female, *M*_*Age*_ = 26.53, range: 19–38 years) were given the identical task but performed it in an fMRI scanner. One participant from Experiment 2 was excluded from all the analyses due to a temporal lobe lesion. All other participants were native German speakers, reported no dyslexia, neurological or psychiatric diseases. The subjects received course credit or were paid in cash for their participation. The data have not been made publicly accessible before but are now available in the file observed_data.RData (in the folder R_Analyses at https://doi.org/10.17605/OSF.IO/MWSED).

### Stimuli and task

The lexical decision task used 400 pairs of stimuli, 200 word−word pairs and 200 word − non-word pairs. We do not analyze the non-word pairs and refer the interested readers to the original studies for more information regarding them.

The words contained three to eight letters, had a Leipzig word frequency class seven to 15^29,109^ and maximally seven orthographic neighbors^10^. Primes and targets in all conditions were matched on length, frequency class, and the number of orthographic neighbors. The word−word pairs were then assigned to eight study conditions. These were formed by combining three experimental factors: the strength of direct association (strong vs. no), the number of common associates (many vs. no) and the SOA (long [1000 ms] vs. short [200 ms]). Associative links were defined to be present, when two words were more likely to co-occur in the sentences of a large text corpus than predictable by their single-word frequencies^29^. If a significant associative link was present (*p <* 0.01), the association strength was set to the log-transformed *χ*^2^ value of the log likelihood test^110^.The strength of direct association was matched between all prime-target pairs in all conditions with a strong direct association. Likewise, the number of common associates (i.e., semantic similarity) was matched across all conditions with many common associates.

The experimental task started with 20 practice trials, which were constructed in the same manner as the main experiment. The viewing distance was about 70 cm, with stimuli displayed in black Times New Roman font on a white background. The stimuli were presented in a pseudo-randomized order with no more than three consecutive pairs of word or non-word targets. A trial started with the presentation of the prime (150 ms), which was followed by a 50 ms (short SOA) or 850 ms (long SOA) blank screen. Next, the target was displayed for 200 ms, followed by eight hash characters (########), which were displayed for a maximum of 1300 ms. Then, the fixation cross (+) appeared for 1–10 s. The participants could respond as soon as the target was displayed and until the fixation cross appeared, resulting in a 1500 ms response window.

#### SROM’s lexicon

To model this task, the SROM uses a lexicon of 9530 German nouns, which had three to nine letters and a Leipzig frequency class between four and 24. Further, they had between one and 1655 significantly associated words in the lexicon.

### The leaky accumulators of the SROM’s decision layer

The SROM contains a two-unit decision layer, where one unit represents the yes response (word unit) and the other the no response (non-word unit). The input into the word unit is the target’s current orthographic activation, transformed using the sigmoid function: $\:{\upsigma\:}\left({a}_{orth{o}_{t}}\right)=\frac{1}{1+\mathrm{exp}\left({a}_{orth{o}_{t}}\right)}$. The non-word decision unit is driven by the lack of a *word identification signal*, which is equal to $\:1-\sigma\:\left({a}_{orth{o}_{t}}\right)$, The processing in the decision layer is governed by the non-linear LCA equations and its parameters^39^(see supplementary Tables 3 and Fig. 2):

1 $$da_{{i_{t} }} = \left( {I_{{i_{t} }} - {{\kappa }} \cdot a_{{i_{{t - 1}} }} - {{\beta }} \cdot {{\sigma }}\left( {a_{{j_{{t - 1}} }} } \right)} \right) \cdot \frac{{dt}}{{{\tau }}} + {{\xi }} \cdot \sqrt {\frac{{dt}}{{{\tau }}}},$$

where the change in activation of unit *i* (*i* ∈ {*word*,* non*-*word*}) at time *t* ($\:d{a}_{{i}_{t}}$) is the sum of a net input and a noise term. The *net input* for unit *i*: $\left( {I_{{i_{t} }} - {{\kappa }} \cdot a_{{i_{{t - 1}} }}-{{\beta }} \cdot {{\sigma }}\left( {a_{{j_{{t - 1}} }} } \right)} \right)$ is given by the:

- sum of the current input from the orthographic layer $\:{I}_{{i}_{t}}$,
- the activation loss due to the leakage of the *i*^*th*^ unit’s activation (${{{\kappa }} \cdot a_{{i_{{t - 1}} }} }$), which is determined by the parameter *κ* and the activation level in the previous cycle $\:{a}_{{i}_{t-1}}$,
- the lateral inhibition from the other decision unit *j* ${{{\beta }} \cdot {{\sigma }}\left( {a_{{j_{{t - 1}} }} } \right)}$, whose strength is given by the product of the lateral inhibition parameter *β* and the sigmoid-transformed activation of the decision unit *j* ($\:{a}_{{j}_{t-1}}$), and.
- by the time scaling constant $\:\frac{dt}{{\uptau\:}}$.

The second term in Eq. 1 is the product of the evidence accumulation *noise ξ* and the square root $\:\sqrt{\frac{dt}{{\uptau\:}}}$ of the scaling constant. *ξ* is the standard deviation of the normal distribution from which a random value is drawn at each processing cycle *t* and added to the decision evidence.

The range of the possible activations of the decision units was limited to the interval [0, 1]. The time scaling constant was 0.05, which means that one processing cycle corresponds to 50 ms. The simulated RTs are then calculated as: $RT = NDT + 0.05 \cdot \:t_{{decision}}$, with *NDT* being the non-decision time and *t*_*decision*_ the processing cycle in which the accumulation process stopped.

#### The time varying decision threshold

The SROM uses a time varying decision threshold, motivated by findings that its value can be increased or decreased by the ongoing processing of the presented stimulus^86,88,111^. In the SROM, the initial decision threshold (*θ*_*initial*_) is freely estimated for each participant. At each processing cycle, the *θ*_*initial*_ is decreased according to the global lexical-semantic activation, which represents a sense of familiarity^54,111^ or a word-likeness signal^96^. For each target, this signal is the product of the global associative (GAA) and the global orthographic activation (GOA). The GAA is based on the current activation values of all active unit pairs ($\:{a}_{i},\:{a}_{j}$) (cf.^54^). It is the weighted sum of the products of the activations, where the weights are the connection strengths between the two units ($\:{w}_{i,j}$), i.e., $\:GAA={\sum\:}_{i}{\sum\:}_{j}{a}_{i}\cdot\:{a}_{j}\cdot\:{w}_{i,j}$. Thus, the GAA corresponds to the Hopfield energy in the associative layer^54^.The GOA is calculated analogously using the currently active orthographic units’ activations (i.e., $\:GOA\:=\:{\sum\:}_{i}{a}_{i}\cdot\:{a}_{i}$). Unlike GAA, the GOA is an unweighted sum because of the strong mutual inhibition in the orthographic layer, which prevents most units from surpassing the activation threshold, effectively reducing the contributions of competing stimuli to zero (e.g., see Fig. 6 in^12^). Formally, the decision threshold at cycle *t* (*θ*_*t*_) is calculated as:

2 $$\theta \:_{t} = \theta \:_{{initial}} \cdot \:{\mathrm{exp}}\left( { - GAA_{t} \cdot \:GOA_{t} } \right)$$

where the global associative activation at time *t* ($\:{GAA}_{t}$) equals the weighted dot product of the activation values of the active associative units: $\:{x}_{t}^{\mathsf{T}}W{x}_{t}$. The vector $\:{x}_{t}$ is the column vector of the activation values of all active units in the associative layer and $\:{x}_{t}^{T}$ is its transpose. $\:W$ is the matrix of the pairwise association strengths. The orthographic global activation ($\:{GOA}_{t}$) equals $\:{y}_{t}^{\mathsf{T}}I{y}_{t}$, where $\:{y}_{t}$ are the current activations of all active orthographic units and *I* is the identity matrix. Finally, the multiplication of the initial decision threshold (*θ*_*initial*_) with $\:\mathrm{e}\mathrm{x}\mathrm{p}\left(-{GAA}_{t}\cdot\:{GOA}_{t}\right)$ causes its decay towards zero (see Fig. 2). Figure 2 demonstrates that the decision threshold behaves as a kind of temporal deadline, which was one of the decision thresholds in the MROM^10^.

Since the semantic priming effect also reflects strategic processes^7^, we expected that the SOA duration would have an effect on the decision layer parameters. That is, the SOA should affect the participants’ ability to use the information provided by the prime in anticipating the potential targets and their expected activation level. To account for this, we use separate decision thresholds (*θ*), leak (*κ*) and inhibition (*β*) parameters for the long and short SOA conditions. For the list of the SROM’s fixed parameters see supplementary Tables 1 and 2 (for the model equations, the reader is referred to the cited references). An overview of the SROM’s free parameters is provided in supplementary Table 3.

#### Estimation of the SROM’s parameters

The model parameters were estimated for each participant using the *’patternsearch’* function in MATLAB (R2023a, Mathworks, Inc., Natick, MA). The details of the optimization are given in Appendix A.

### fMRI data and processing

### MRI Task

The MRI task was the same as in the behavioral experiment.

### MRI data acquisition

The MRI data were acquired at the Center for Cognitive Neuroscience Berlin in a Siemens Magnetom 3 T TrioTim Syngo MR B17 scanner with a 12 channel receiver head coil. Stimuli were projected to a mirror above the participant’s head. Prior to scanning, shimming was performed. Functional (T2*) and structural (T1) scans were aligned to AC/PC line. 37 slices of functional data were acquired in descending order using echo planar imaging (scanning parameters: TR 2 s, TE 30 ms, in-plane resolution 3 × 3 mm, FOV 192 mm, matrix size 64 × 64 mm). The 176 T1 slices were also acquired in descending order and with the following parameters: TR 1900 ms, TE 2.52 ms, in-plane resolution 1 × 1 mm, FOV 256 mm, matrix size 256 × 256 mm.

## MRI preprocessing

MRI analyses were performed in MATLAB R2023a using SPM12 with the default preprocessing settings. First, motion correction was applied by realigning functional data to the mean functional image. Next, slice timing correction was applied, using the middle (19th ) slice as reference. The structural image was then coregistered to the functional scans and segmented into four tissue types. In the next step, the structural scans were normalized to the SPM Montreal Neurological Institute (MNI) T1 template. The normalization parameters of the structural scan were then used to align the functional images with the MNI template. Finally, the functional scans were resampled to isotropic voxel size of [2 × 2 × 2 mm] and smoothed using an 8 mm FWHM Gaussian kernel.

### MRI statistical analyses

First-level fixed effects analyses were performed using a 1/128 Hz high-pass temporal filter. The analyses were performed using a first order autoregressive model and the canonical hemodynamic response function with its temporal derivatives. The effects of interest were the brain responses to the presentations of target words from each experimental condition. Following SPM’s recommendations all events were modeled with delta functions, convolved with the canonical hemodynamic response function. The presentation of zero events (e.g., fixation crosses) served as baseline.

To investigate the neural correlates of the word identification signal, i.e., the source of the decision evidence in the SROM, each target was also assigned a parametric modulator. Its value was the mean centered target’s maximum orthographic activation predicted by the SROM. Before mean centering, the orthographic activation values were transformed using the sigmoid function. The first-level models further contained the following variables of no interest: the six estimated movement parameters, the presentation of non-words and targets, which were not recognized as words, zero events, button presses and the presentation of primes.

In the second-level random effects analysis, we specified a full factorial model with three factors and five second-level covariates. The factors were the SOA (long, short), the direct association strength (strong, none) and semantic similarity, i.e., the number of common associates (many, few). The second-level covariates were the participants’ estimated and normalized SROM’s free parameters: decision threshold (*θ*), leak (*κ*), mutual inhibition (*β*), evidence accumulation noise (*ξ*) and the strength of the excitation from the associative to orthographic word unit ($\:{\alpha\:}_{ao}$). Since the values of the *θ*, *κ* and *β* parameters depended on the SOA, these covariates interacted with the SOA factor. Due to high collinearity between the decision threshold, leak and inhibition parameters, we had to combine the leak and mutual inhibition parameter into a single parameter *λ* = *κ* – *β*, called effective differential leakage^39^. It is possible to fit the LCA using just the differential leakage, which reduces the LCA to the Ornstein-Uhlenbeck process and can be used to model data from tasks, where participants must make a yes/no decision^39^. *λ* measures the balance between self-inhibition and mutual inhibition^39,112^. When greater than zero (*κ* > *β*), the decision process is slowed down, because of stronger self-inhibition of all decision units; when *λ* is less than zero, the decision process is speeded in favor of the preferred alternative, because the decision unit with the strongest input and highest activation inhibits competing units more strongly than it is losing activation (i.e., *κ* < *β*)^112^. The final model thus contained three factors and four covariates each interacting with the SOA. The effects of the SROM’s parameters across both SOA levels were analyzed using F-tests to simultaneously test for significant increases and decreases of the BOLD response.

The second-level analysis for the SROM’s free parameters was constrained to a regions-of-interest mask containing the following left hemisphere regions AAL3 regions^113^: the inferior frontal gyrus (partes orbitalis, opercularis, triangularis), the supplementary motor area, the inferior and middle temporal gyrus, the fusiform and lingual gyri as well as the angular gyrus. Statistical inference was performed at the voxel level using $\:{p}_{FWE}<0.05.$ We report clusters containing at least 60 voxels.

A separate second-level analysis examined the effect of the maximum orthographic activation. In this analysis the first-level parametric modulator contrasts from each subject were submitted to an F-test, again simultaneously testing for both increased and decreased BOLD responses. Following Hofmann and Jacobs^3^ and the studies cited therein, this analysis was constrained to a regions-of-interest mask consisting of the AAL3 left fusiform, lingual, inferior and middle temporal gyri. Statistical inference was performed at the voxel level using $\:{p}_{FWE}<0.05.$.

### Statistical analyses

Statistical analyses were performed in R^114^. We used the following packages: *lme4*, *emmeans*,* rstatix* and *correlations*. Figures were created using the *ggplot2* and *patchwork* packages as well as MATLAB and Krita software.

To assess the global correspondence between observed and simulated data, we correlated the simulated and observed average response times (RT) and mean accuracy rates across both experiments and all conditions. Next, observed and simulated data were compared using correlations at the level of the conditional means of each participant, i.e., the level at which the model was fitted to the data. All analyses were performed on RTs for correct responses, as customary in semantic priming research. We first calculated the mean and standard deviations of the RTs and error proportions of the eight experimental conditions. Then, we calculated the Spearman correlations to assess how well simulated RTs and error proportions correspond to observed data. We used Bonferroni correction for multiple comparisons. For visual comparison of RTs, we also plotted the empirical cumulative distribution functions of simulated and observed RT, shown in supplementary Fig. 1.

To further investigate the correspondence between simulated and observed data, we calculated the priming effects for RTs and error proportions of the three primed conditions: associative (strong direct association and few common associates), semantic (no direct association and many common associates) and combined (strong direct association and many common associates). The average RTs and error proportions were subtracted from the average RTs and error proportions for targets with no direct association and few common associates with the prime. This was done separately for each Experiment and SOA. Finally, the priming effects were tested against no effect for each priming condition (associative, semantic, combined) in observed and simulated data.

To investigate the relationships between the free parameters of the SROM, we calculated the pairwise correlations between them in each SOA condition. For the initial decision threshold (*θ*), leak (*κ*) and mutual inhibition (*β*) parameters paired Wilcoxon tests were used to investigate if their values differed between the two SOA conditions. For Wilcoxon tests the r effect size measure is reported. To explore the relationship between the participants’ model parameters and their behavioral data, we also calculated the correlations between the free parameters, mean RTs and accuracy. Concretely, we first calculated average RTs and accuracy for each level of SOA in each experiment and then calculated their correlations with the decision threshold (*θ*), leak (*κ*) and mutual inhibition(*β*) parameters. Since the non-decision time, the evidence accumulation noise (*ξ)* and the associative to orthographic excitation strength (*α*_*ao*_) did not vary with SOA, the correlations were calculated using the overall mean RTs and accuracy.

## Supplementary Information

Below is the link to the electronic supplementary material.

Supplementary Material 1

## Data Availability

Data, simulation and analysis code are available on Open Science Framework (https:/doi.org/10.17605/OSF.IO/MWSED).
