# Supplementary material for: A neurocognitive interactive activation model of semantic priming in lexical decisions
Source: Sci Rep. 2026 Jun 20;16:19183. doi: 10.1038/s41598-026-58866-4 (PMC13283216; doi:10.1038/s41598-026-58866-4)
Supplement: Supplementary file 1 — Supplementary Material 1 [file 41598_2026_58866_MOESM1_ESM.pdf]

A neurocognitive interactive activation model of semantic priming in lexical decisions: Supplementary Information

Leo Sokolovič[1, 2, 3, *], Juraj Kukolja[2, 3], Markus Hofmann[1]

Contents

Model parameters 2
Optimization 4
Simulated and observed response times and error proportions 6
Comparison of empirical cumulative density functions 8
Experiment 1: Correlations 9
Experiment 2: Correlations 12
Cosine similarities between predictors 14

Affiliations:

[1] Department of General and Biological Psychology, University of Wuppertal, 42119 Wuppertal, Germany

[2] Department of Neurology and Clinical Neurophysiology, Helios University Hospital Wuppertal, 42883 Wuppertal, Germany

[3] Faculty of Health, Witten/Herdecke University, 58488 Witten, Germany

* corresponding author

Model parameters

Supplementary table 1. All excitation and inhibition model parameters of the SAROM

Excitation parameter α					
Layer	Feature	Letter	Orthographic	Associative	Response
Feature	0	0.005	0	0	0
Letter	0	0	0.07	0	0
Orthographic	0	0.3	0	0.09	0
Associative	0	0	α_{ao}	0.03	1
Response	0	0	0	0	0
**Inhibition parameter γ**					
Layer	Feature	Letter	Orthographic	Associative	Response
Feature	0	-0.15	0	0	0
Letter	0	0	-0.04	0	0
Orthographic	0	0	-0.21	0	0
Associative	0	0	0	-0.04	0
Response	0	0	0	0	β

Note: β parameter of the LCA was estimated from data

Supplementary table 2. Decay parameters

Layer	SAROM
Feature	0.00
Letter	0.07
Orthographic	0.07
Associative	0.07
Response	κ

Note: κ parameter of the LCA was estimated from data

Supplementary table 3. SROM's free parameters and their mean fitted values for short and long SOA

			short SOA		long SOA	
Parameter	Function	Range	*M*	*SD*	*M*	*SD*
leak parameter (κ)	the proportion of the unit's activation lost in each cycle	(0, 1)	0.85	0.16	0.69	0.25
mutual inhibition (β)	the strength of the mutual inhibition between the decision units	(0, 1)	0.29	0.23	0.16	0.15
decision threshold (θ)	the amount of evidence needed for a decision	(0, 1]	0.13	0.06	0.18	0.06
evidence accumulation noise (ξ)	a random, nonspecific input to the decision units	(-∞, ∞)	0.08	0.03	0.08	0.03
associative to orthographic excitation (α_{ao})	the proportion of the target's associative activation sent to its orthographic unit	(0.01, 0.12)	0.06	0.04	0.06	0.04
non-decision time (*NDT*)	the part of the response time due to non-cognitive processes	(0, RT_{min}]	0.24	0.11	0.24	0.11

Note: RT_{min}: the subject's shortest response time. Average parameter values for each experiment are reported in the supplementary table 3.

Supplementary table 4. Mean parameter values

	Experiment 1				Experiment 2			
	SOA: short		SOA: long		SOA: short		SOA: long	
Parameter	M	SD	M	SD	M	SD	M	SD
leak parameter	0.84	0.18	0.71	0.27	0.85	0.14	0.68	0.24
mutual inhibition	0.30	0.24	0.14	0.15	0.28	0.23	0.17	0.16
decision threshold	0.13	0.06	0.18	0.06	0.13	0.05	0.18	0.07
evidence accumulation noise	0.08	0.03	0.08	0.03	0.07	0.02	0.07	0.02
effective differential leakage	0.54	0.24	0.56	0.26	0.57	0.23	0.51	0.23
associative to orthographic excitation	0.06	0.04	0.06	0.04	0.05	0.04	0.05	0.04
non-decision time	0.26	0.11	0.26	0.11	0.23	0.11	0.23	0.11

Optimization

We fitted the SROM in MATLAB using the following cost function, which is based on the one used by Usher and McClelland (21):

$$C = 100 \cdot \sum_{i=1}^{8} \frac{\left(\langle RT \rangle_{i,observed} - \langle RT \rangle_{i,simulated}\right)^2}{SD_{i,observed}^2} +$$
$$\sum_{i=1}^{8} \left(100 \cdot \left[SD_{i,observed} - SD_{i,simulated}\right]\right)^2 +$$
$$\sum_{i=1}^{8} \left(100 \cdot \left[PC_{i,observed} - PC_{i,simulated}\right]\right)^2 +$$
$$\sum_{i=1}^{7} \sum_{j=i+1}^{8} \left(100 \cdot \left[\Delta_{i,j,observed} - \Delta_{i,j,simulated}\right]\right)^2, \quad (3)$$

where SD_i is the standard deviation of response times of correct responses in condition i and PC_i is the proportion of correct responses in condition i. The $\Delta_{i,j}$ in the final line of equation 3 is $\Delta_{i,j} = \langle RT \rangle_i - \langle RT \rangle_j$, where $\langle RT \rangle_i$ is the average response time of correct responses in condition i and $\langle RT \rangle_j$ the average response time of correct responses in condition j. The experimental conditions correspond to the eight cells of the factorial design combining the three factors SOA (long vs. short), direct association (strong vs. no) and the number of common associates (many vs. few), i.e., semantic similarity. The differences in mean response times of correct responses were calculated between all pairs of conditions and stored in an upper triangular matrix. We calculate one Δ matrix for the observed and one for the simulated data. In the final line of the cost function (equation 3), we calculate a sum of the squared differences between individual cells of the observed and simulated Δ matrices. These matrices were calculated to aid model fitting, which was necessary due to a very low number of trials per person per condition (25 if all trials were correct). Normally, sequential sampling models require more than 100 trials per condition to achieve reasonable fits [1,2]. As in Usher and McClelland [3] the multiplication by 100 balances the contributions of response times, their standard deviations and the accuracy to the total cost.

Since all SROM layers - apart from the decision layer - are deterministic and there is no feedback from the decision layer to the preceding layers, the model was fitted in two steps. First, we simulated 12 versions of the SROM, one for each α_{ao} level. Next, the decision layer parameters were optimized for each participant at each value of the excitation strength from the associative to the orthographic layer (α_{ao}). The final model was the model with the smallest cost.

The optimization was run using MATLAB's '*patternsearch*' algorithm with the following settings: Algorithm: '*classic*', PollMethod: '*GSSPositiveBasis2N*', PollOrderAlgorithm: '*Success*', SearchFcn: '*rbfsurrogate*', TolFun: '*0.001*', UseCompletePoll: '*true*', Cache: '*off*', UseParallel: '*true*'. All other settings were at their default values.

References

1. Miletić, S., Turner, B. M., Forstmann, B. U. & van Maanen, L. Parameter recovery for the Leaky Competing Accumulator model. *J. Math. Psychol.* **76**, 25–50 (2017).
2. Ratcliff, R. & Tuerlinckx, F. Estimating parameters of the diffusion model: Approaches to dealing with contaminant reaction times and parameter variability. *Psychon. Bull. Rev.* **9**, 438–481 (2002).
3. Usher, M. & McClelland, J. L. The time course of perceptual choice: The leaky, competing accumulator model. *Psychol. Rev.* **108**, 550–592 (2001).

Simulated and observed response times and error proportions

Supplementary table 5. Observed and simulated response times in seconds

			observed		simulated			
SOA	Direct association	Common associates	M	SD	M	SD	ρ	p_{Bonf}
			Experiment 1					
long	strong	many	0.65	0.15	0.64	0.15	0.94	< 0.001
long	strong	few	0.66	0.16	0.66	0.16	0.88	< 0.001
long	no	many	0.68	0.17	0.68	0.17	0.87	< 0.001
long	no	few	0.69	0.16	0.67	0.16	0.91	< 0.001
short	strong	many	0.68	0.15	0.70	0.15	0.94	< 0.001
short	strong	few	0.71	0.16	0.70	0.16	0.89	< 0.001
short	no	many	0.71	0.16	0.70	0.15	0.91	< 0.001
short	no	few	0.72	0.16	0.71	0.16	0.86	< 0.001
			Experiment 2					
long	strong	many	0.63	0.15	0.63	0.15	0.93	< 0.001
long	strong	few	0.65	0.17	0.66	0.17	0.93	< 0.001
long	no	many	0.69	0.18	0.67	0.17	0.96	< 0.001
long	no	few	0.70	0.17	0.68	0.18	0.95	< 0.001
short	strong	many	0.65	0.14	0.66	0.14	0.93	< 0.001
short	strong	few	0.68	0.15	0.68	0.15	0.93	< 0.001
short	no	many	0.68	0.18	0.67	0.16	0.94	< 0.001
short	no	few	0.71	0.19	0.69	0.17	0.95	< 0.001

Note: df $\rho_{Exp.1}$ = 30, df $\rho_{Exp.2}$ = 29

Supplementary table 6. Observed and simulated error proportions

			observed		simulated			
SOA	Direct association	Common associates	M	SD	M	SD	ρ	p_{Bonf}
			Experiment 1					
long	strong	many	0.04	0.05	0.04	0.04	0.69	< 0.001
long	strong	few	0.06	0.05	0.05	0.06	0.72	< 0.001
long	no	many	0.07	0.07	0.06	0.07	0.73	< 0.001
long	no	few	0.05	0.07	0.05	0.08	0.88	< 0.001
short	strong	many	0.03	0.04	0.03	0.05	0.86	< 0.001
short	strong	few	0.05	0.06	0.05	0.06	0.83	< 0.001
short	no	many	0.07	0.07	0.06	0.07	0.83	< 0.001
short	no	few	0.08	0.07	0.07	0.07	0.90	< 0.001
			Experiment 2					
long	strong	many	0.03	0.04	0.03	0.03	0.46	0.008
long	strong	few	0.06	0.06	0.05	0.05	0.78	< 0.001
long	no	many	0.06	0.05	0.05	0.05	0.77	< 0.001
long	no	few	0.06	0.05	0.06	0.05	0.78	< 0.001
short	strong	many	0.03	0.04	0.03	0.05	0.59	0.005
short	strong	few	0.04	0.05	0.04	0.05	0.73	< 0.001
short	no	many	0.05	0.04	0.04	0.05	0.80	< 0.001
short	no	few	0.06	0.04	0.05	0.04	0.78	< 0.001

Note: df $\rho_{Exp.1}$ = 30, df $\rho_{Exp.2}$ = 29

Comparison of empirical cumulative density functions

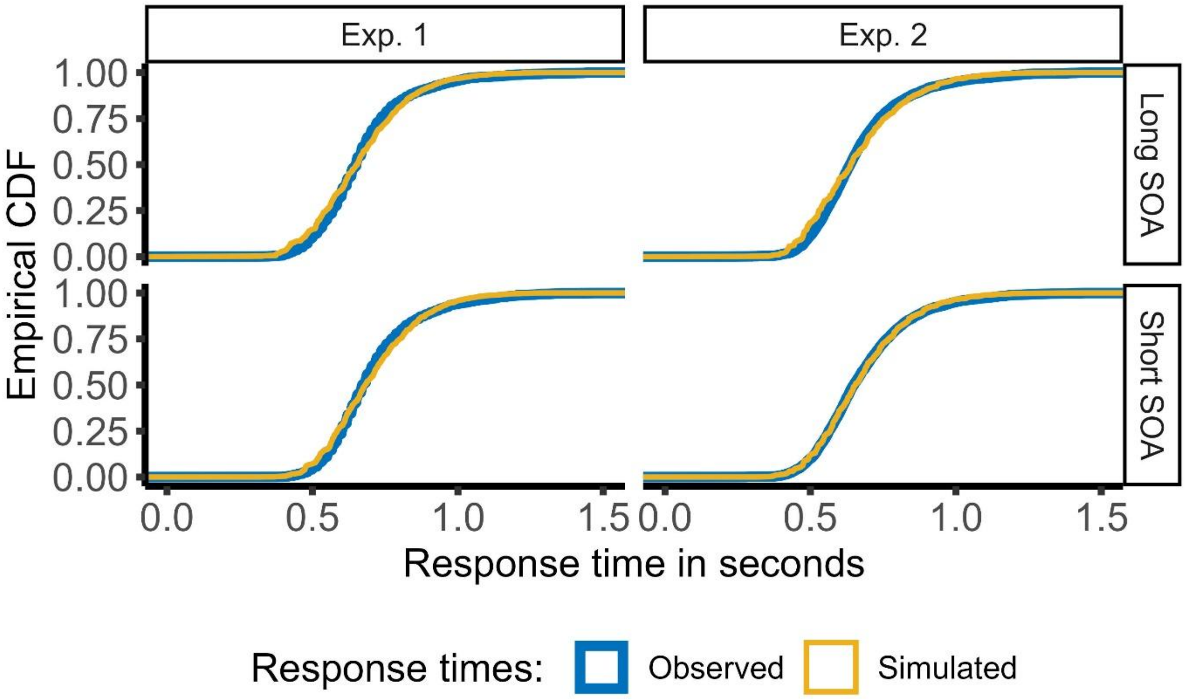

Supplementary Figure 1. The plots of the empirical cumulative density functions (eCDF) show that the distributions of the simulated and observed response times (RT) are similar. This demonstrates that extending the underlying interaction activation model with leaky competing accumulators enables it to reproduce the skewed, non-normally distributed response times. Long and short stimulus-onset-asynchrony (SOA) RT from both experiments (Exp.) are plotted, because the decision parameters were only allowed to vary with the SOA.

Experiment 1: Correlations

The following tables list correlations between SAROM's free parameters and their correlations with the mean response times and error rates.

Supplementary table 7. Correlations in the short SOA

Parameters	ρ	95% CI	S	p_{bonf}
κ & β	0.30	[-0.07, 0.59]	3842.15	> 0.999
κ & ξ	0.19	[-0.52, 0.18]	6509.65	> 0.999
κ & NDT	0.35	[0.00, 0.63]	3522.62	0.699
**κ & θ**	**-0.64**	**[-0.81, -0.37]**	8960.76	0.001
κ & α_{AO}	0.41	[0.07, 0.67]	3198.45	0.278
κ & RT	-0.20	[-0.52, 0.17]	6529.88	> 0.999
κ & PC	0.27	[-0.09, 0.58]	3965.13	> 0.999
**β & ξ**	**-0.81**	**[-0.91, -0.64]**	9874.00	< 0.001
β & NDT	-0.24	[-0.55, 0.13]	6750.00	> 0.999
**β & θ**	**-0.77**	**[-0.89, -0.57]**	9672.00	< 0.001
β & α_{AO}	0.22	[-0.15, 0.54]	4241.25	> 0.999
β & RT	-0.16	[-0.49, 0.21]	6354.00	> 0.999
β & PC	0.33	[-0.03, 0.62]	3653.07	> 0.999
**ξ & θ**	**0.65**	**[0.39, 0.82]**	1884.00	< 0.001
ξ & λ	0.61	[0.33, 0.80]	2106.00	0.004
NDT & θ	-0.23	[-0.54, 0.14]	6698.00	> 0.999
NDT & λ	0.50	[0.17, 0.73]	2728.00	0.075
**θ & α_{AO}**	**-0.58**	**[-0.78, -0.28]**	8613.53	< 0.001
θ & λ	0.29	[-0.08, 0.59]	3886.00	>0.999
θ & RT	0.39	[0.03, 0.65]	3346.00	0.604
θ & PC	-0.22	[-0.53, 0.15]	6646.20	> 0.999
α_{AO} & λ	0.13	[-0.24, 0.46]	4760.27	> 0.999

Note: significant correlations are marked in bold. NDT: non-decision time; κ: leak parameter; β: mutual inhibition; θ: decision threshold; ξ: evidence accumulation noise; α_{AO}: associative-orthographic excitation strength, λ: effective differential leakage; RT: response time; PC: proportion correct

Supplementary table 8. Correlations in the long SOA

Parameters	ρ	95% CI	S	p_{bonf}
κ & β	0.29	[-0.07, 0.59]	3867.40	> 0.999
κ & ξ	0.41	[0.06, 0.67]	3206.94	0.286
κ & NDT	0.44	[0.10, 0.69]	3059.80	0.179
**κ & θ**	**-0.70**	**[-0.84, -0.45]**	9256.48	< 0.001
κ & α_{AO}	0.20	[-0.17, 0.52]	4359.91	> 0.999
κ & RT	-0.00	[-0.36, 0.35]	5481.02	> 0.999
κ & PC	0.34	[-0.03, 0.62]	3622.86	> 0.999
β & ξ	-0.07	[-0.42, 0.30]	5828.03	> 0.999
β & NDT	-0.00	[-0.36, 0.35]	5496.00	> 0.999
β & θ	-0.44	[-0.69, -0.10]	7862.22	0.173
β & α_{AO}	-0.15	[-0.48, 0.22]	6262.88	> 0.999
β & RT	0.06	[-0.30, 0.41]	5118.97	> 0.999
β & PC	-0.00	[-0.36, 0.36]	5462.04	> 0.999
ξ & NDT*	0.40	[0.05, 0.66]	3276.00	0.235
ξ & θ	-0.04	[-0.39, 0.32]	5676.00	> 0.999
ξ & α_{AO}*	-0.05	[-0.40, 0.31]	5742.77	> 0.999
ξ & RT*	0.47	[0.14, 0.71]	2878.00	0.063
ξ & PC*	-0.47	[-0.71, -0.13]	8004.31	0.070
ξ & λ	0.61	[0.32, 0.79]	2138.00	0.005
**NDT & θ**	**-0.56**	**[-0.76, -0.25]**	8486.00	0.015
NDT & λ	0.40	[0.05, 0.66]	3290.00	0.514
**NDT & α_{AO}***	**0.66**	**[0.40, 0.82]**	1841.05	< 0.001
NDT & RT*	0.38	[0.03, 0.65]	3358.00	0.298
NDT & PC*	-0.33	[-0.62, 0.03]	7254.46	0.654
**θ & α_{AO}**	**-0.45**	**[-0.70, -0.11]**	1841.05	< 0.001
θ & RT	0.22	[-0.15, 0.54]	4262.00	> 0.999
θ & PC	-0.13	[-0.47, 0.24]	6187.98	> 0.999
θ & λ	-0.40	[-0.66, -0.05]	7624.00	0.511
α_{AO} & RT*	-0.03	[-0.39, 0.33]	5636.75	> 0.999
α_{AO} & PC*	-0.17	[-0.49, 0.21]	6357.43	> 0.999
α_{AO} & λ	0.17	[-0.20, 0.50]	4512.88	> 0.999

Note: significant correlations are marked in bold. NDT: non-decision time; κ: leak parameter; β: mutual inhibition; θ: decision threshold; ξ: evidence accumulation noise; α_{AO}: associative-orthographic excitation strength; RT: response time; PC: proportion correct; correlations marked with * were calculated for RT and PC averaged over all the experimental factors

Experiment 2: Correlations

The following tables list correlations between SAROM's free parameters and their correlations with the mean response times and error rates.

Supplementary table 9. Correlations in the short SOA

Parameters	ρ	95% CI	S	p_{bonf}
κ & β	0.30	[-0.07, 0.60]	3485.02	> 0.999
κ & ξ	-0.16	[-0.49, 0.22]	5729.55	> 0.999
κ & NDT	0.37	[0.01, 0.65]	3122.29	0.603
**κ & θ**	**-0.64**	**[-0.82, -0.36]**	8150.44	0.001
**κ & α_{AO}**	**0.52**	**[0.19, 0.74]**	2372.13	0.039
κ & RT	-0.21	[-0.53, 0.17]	6008.12	> 0.999
κ & PC	0.26	[-0.12, 0.57]	3676.21	> 0.999
β & ξ	-0.40	[-0.66, -0.04]	6924.00	0.412
β & NDT	0.00	[-0.36, 0.37]	4944.00	> 0.999
**β & θ**	**-0.62**	**[-0.80, -0.33]**	8032.00	0.003
β & α_{AO}	0.40	[0.04, 0.67]	2973.11	0.383
β & RT	0.18	[-0.19, 0.51]	4056.00	>0.999
β & PC	-0.39	[-0.66, -0.04]	6918.65	0.586
ξ & θ	0.49	[0.15, 0.72]	2546.00	0.082
ξ & λ	0.30	[-0.07, 0.60]	3478.00	> 0.999
NDT & θ	0.14	[-0.24, 0.48]	4289.66	> 0.999
NDT & λ	0.25	[-0.12, 0.56]	3706.00	> 0.999
**θ & α_{AO}**	**-0.70**	**[-0.85, -0.44]**	8409.58	< 0.001
θ & λ	0.24	[-0.13, 0.56]	3758.00	>0.999
θ & RT	0.27	[-0.11, 0.58]	3630.00	> 0.999
θ & PC	0.21	[-0.16, 0.54]	3898.90	> 0.999
α_{AO} & λ	-0.04	[-0.40, 0.33]	5160.60	> 0.999

Note: significant correlations are marked in bold. NDT: non-decision time; κ: leak parameter; β: mutual inhibition; θ: decision threshold; ξ: evidence accumulation noise; α_{AO}: associative-orthographic excitation strength; λ: effective differential leakage; RT: response time; PC: proportion correct

Supplementary table 10. Correlations in the long SOA

Parameters	ρ	95% CI	S	p_{bonf}
κ & β	0.44	[0.09, 0.69]	2771.12	0.194
κ & ξ	0.50	[0.17, 0.73]	2483.00	0.064
κ & NDT	0.31	[-0.06, 0.61]	3399.37	> 0.999
**κ & θ**	**-0.55**	**[-0.76, -0.23]**	7685.10	0.021
κ & α_{AO}	-0.16	[-0.50, 0.21]	5768.79	> 0.999
κ & RT	0.09	[-0.28, 0.44]	4503.82	> 0.999
κ & PC	0.37	[0.00, 0.65]	3136.09	0.878
β & ξ	0.15	[-0.32, 0.49]	4204.00	> 0.999
β & NDT	0.03	[-0.34, 0.39]	4830.00	> 0.999
β & θ	-0.46	[-0.70, -0.11]	7234.00	0.142
β & α_{AO}	-0.13	[-0.47, 0.24]	5610.20	> 0.999
β & RT	0.31	[-0.06, 0.61]	3410.00	> 0.999
β & PC	-0.20	[-0.53, 0.17]	5963.45	> 0.999
ξ & NDT*	0.20	[-0.18, 0.53]	3960.00	> 0.999
ξ & θ	0.02	[-0.35, 0.38]	4876.00	> 0.999
ξ & α_{AO}*	-0.24	[-0.55, 0.14]	6136.41	> 0.999
ξ & λ	0.46	[0.12, 0.71]	2664.00	0.183
ξ & RT*	0.41	[0.05, 0.67]	2639.00	0.227
ξ & PC*	-0.13	[-0.48, 0.24]	5627.22	> 0.999
**NDT & θ**	**-0.54**	**[-0.75, -0.22]**	7634.00	0.026
**NDT & α_{AO}***	**0.63**	**[0.34, 0.81]**	1850.13	0.002
NDT & RT*	0.31	[-0.06, 0.61]	3406.00	0.861
NDT & PC*	-0.16	[-0.49, 0.22]	5750.63	> 0.999
NDT & λ	0.26	[-0.12, 0.57]	3672.00	> 0.999
θ & α_{AO}	-0.20	[-0.53, 0.18]	5956.97	> 0.999
θ & λ	-0.28	[-0.59, 0.09]	6366.00	>0.999
θ & RT	0.26	[-0.12, 0.57]	3680.00	> 0.999
θ & PC	0.10	[-0.27, 0.45]	4462.31	> 0.999
α_{AO} & RT*	0.07	[-0.30, 0.43]	4589.03	> 0.999
α_{AO} & PC*	-0.18	[-0.51, 0.20]	5850.56	> 0.999
α_{AO} & λ	-0.05	[-0.40, 0.32]	5190.85	> 0.999

Note: significant correlations are marked in bold. NDT: non-decision time; κ: leak parameter; β: mutual inhibition; θ: decision threshold; ξ: evidence accumulation noise; α_{AO}: associative-orthographic excitation strength; λ: effective differential leakage; RT: response time; PC: proportion correct; correlations marked with * were calculated for RT and PC averaged over all the experimental factors

Cosine similarities between predictors

Supplementary table 11. SPM cosine similarities between SROM's free parameters

Long SOA				
	κ	β	ξ	α_{AO}
θ	-.69	-.67	.01	-.31
κ	-	.41	.32	.09
β	-	-	-.15	.00
ξ	-	-	-	-.01
**Short SOA**				
	κ	β	ξ	α_{AO}
θ	-.74	-.79	.39	-.39
κ		.45	-.15	.27
β			-.53	.16
ξ				-.01

Supplementary table 12. SPM cosine similarities between SROM's decision threshold, differential effective leak, evidence accumulation noise and associative-to-orthographic excitation

Long SOA			
	λ	ξ	α_{AO}
θ	-.32	.40	-.39
λ	-	.44	.10
ξ	-	-	-.01
Short SOA			
	λ	ξ	α_{AO}
θ	.24	.40	-.39
λ		-.43	.05
ξ			-.01

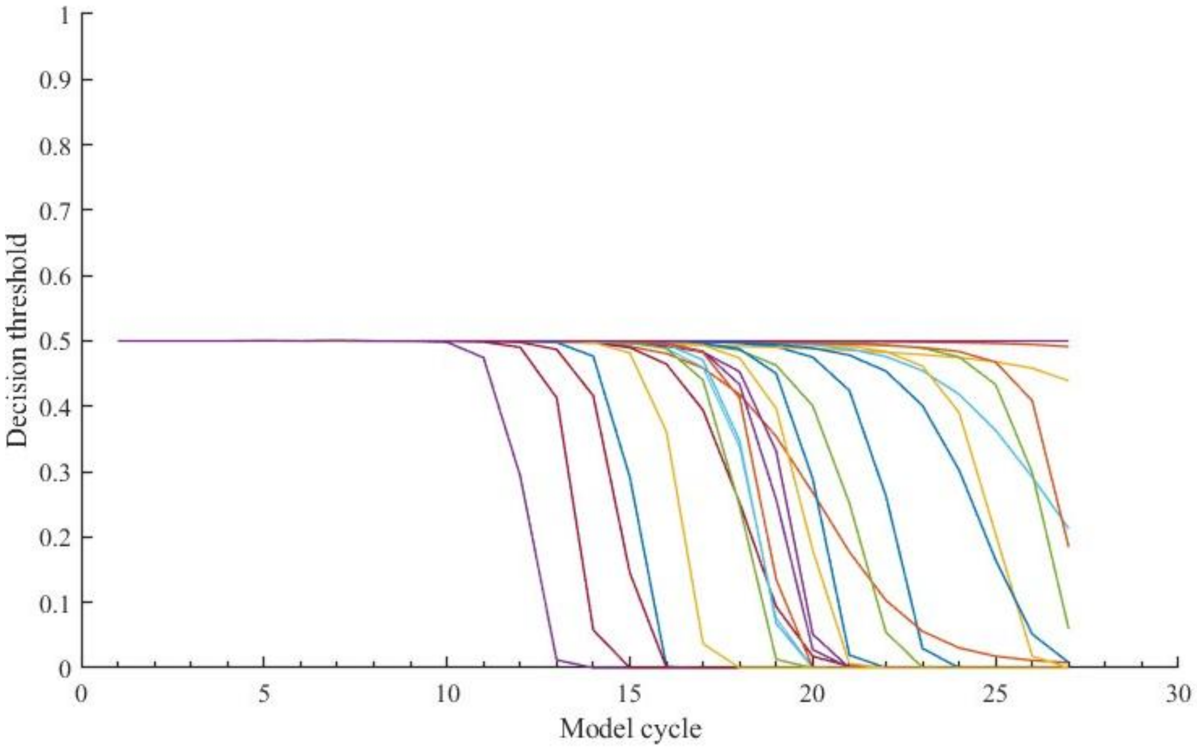

Supplementary Figure 2. Decision threshold time courses for target words from the short SOA, no direct association and no common associates condition. The figure shows that the decision threshold varies across targets despite minimal priming. The initial criterion was arbitrarily set to .5.
